# Omega-6 highly unsaturated fatty acids in Leydig cells facilitate male sex hormone production

**DOI:** 10.1038/s42003-022-03972-y

**Published:** 2022-09-21

**Authors:** Keiken Ri, Hyeon-Cheol Lee-Okada, Takehiko Yokomizo

**Affiliations:** Department of Biochemistry, Juntendo University Graduate School of Medicine, Tokyo, Japan

**Keywords:** Fatty acids, Endocrine reproductive disorders, Lipidomics, Hormone receptors

## Abstract

Highly unsaturated fatty acids (HUFAs) are fatty acids with more than three double bonds in the molecule. Mammalian testes contain very high levels of omega-6 HUFAs compared with other tissues. However, the metabolic and biological significance of these HUFAs in the mammalian testis is poorly understood. Here we show that Leydig cells vigorously synthesize omega-6 HUFAs to facilitate male sex hormone production. In the testis, FADS2 (Fatty acid desaturase 2), the rate-limiting enzyme for HUFA biosynthesis, is highly expressed in Leydig cells. In this study, pharmacological and genetic inhibition of FADS2 drastically reduces the production of omega-6 HUFAs and male steroid hormones in Leydig cells; this reduction is significantly rescued by supplementation with omega-6 HUFAs. Mechanistically, hormone-sensitive lipase (HSL; also called LIPE), a lipase that supplies free cholesterol for steroid hormone production, preferentially hydrolyzes HUFA-containing cholesteryl esters as substrates. Taken together, our results demonstrate that Leydig cells highly express FADS2 to facilitate male steroid hormone production by accumulating omega-6 HUFA-containing cholesteryl esters, which serve as preferred substrates for HSL. These findings unveil a previously unrecognized importance of omega-6 HUFAs in the mammalian male reproductive system.

## Abstract (web summary)

Leydig cells highly express FADS2 to facilitate male steroid hormone production by accumulating omega-6 HUFA-containing cholesteryl esters, which serve as preferred substrates for hormone-sensitive lipase

## Introduction

**Fig. 1:**
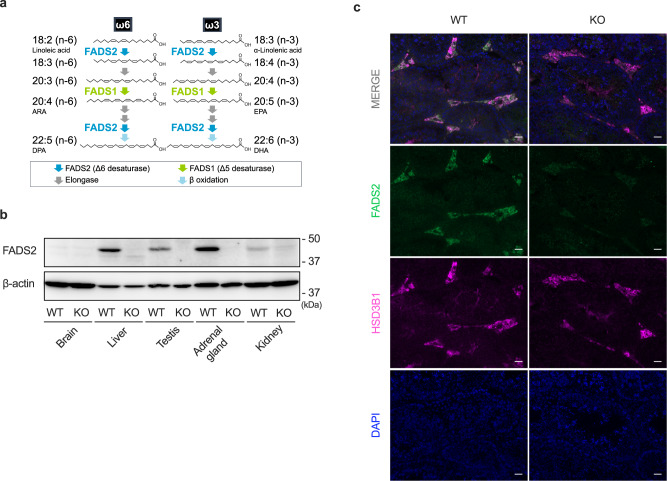
FADS2 is highly expressed in Leydig cells. **a** The biosynthetic pathway of HUFAs. **b** Tissues from FADS2^+/+^ (WT) and FADS2^−/−^ (KO) mice were analyzed by immunoblotting using an anti-mouse FADS2 antibody. β-actin was used as a loading control. **c** Immunohistochemical analysis of testis sections from 12-week-old FADS2^+/+^ and FADS2^−/−^ mice. Green, FADS2; Magenta, HSD3B1; blue, DAPI. Scale bar, 50 µm.

Highly unsaturated fatty acids (HUFAs) are important constituents of biological membranes. HUFAs contain more than three double bonds in the molecule and are classified into omega-6 (n-6) and omega-3 (n-3) HUFAs depending on the position of the first double bond from the methyl terminal carbon. HUFAs are believed to be synthesized from essential fatty acids such as linoleic acid (18:2n-6) and α-linolenic acid (18:3n-3) mainly in the liver, which highly expresses the HUFA synthetic enzymes fatty acid desaturases FADS1 (Fatty acid desaturase 1) and FADS2 (Fatty acid desaturase 2) and elongases ELOVL2 and ELOVL5^1–6^ (Fig. 1a). In many tissues, omega-6 HUFA arachidonic acid (20:4n-6; ARA) and omega-3 HUFA docosahexaenoic acid (22:6n-3; DHA) are the most abundant HUFAs^5^. DHA is highly enriched in the brain and retina and has been shown to be indispensable for the normal function and development of these tissues^1,5–7^. In the testis, omega-6 HUFAs, especially ARA and docosapentaenoic acid (22:5n-6; DPAn-6), are the most abundant HUFAs^5,8^. However, the mechanism, metabolism, and role of omega-6 HUFAs characteristically enriched in the testis remain to be elucidated.

The testis is composed of several cell types. Spermatogenesis occurs within the seminiferous tubules, where spermatozoa develop from germ cells with the support of Sertoli cells that provide structural and metabolic support for the development of sperm cells. Leydig cells are interstitial cells that surround the seminiferous tubules and are widely known to be responsible for producing male steroid hormones. Steroidogenesis is initiated by the release of gonadotrophin-releasing hormone from the hypothalamus and stimulates the release of luteinizing hormone (LH) from the anterior pituitary. LH binds to the G-protein coupled LH receptor expressed in the cell surface of Leydig cells and activates protein kinase A (PKA) signaling. PKA phosphorylates hormone-sensitive lipase (HSL) to liberate cholesterol from cholesteryl esters stored in lipid droplets within the cells^9,10^. Leydig cells have a large number of lipid droplets mainly composed of cholesteryl esters^11^. These cholesteryl esters contain high levels of omega-6 HUFAs, such as DPAn-6, as fatty acids^8,12^; however, how the fatty acyl moiety of cholesteryl esters affects their metabolisms or functions are unknown.

In this study, we used a FADS2^−/−^ mouse model^13^ and an anti-mouse FADS2 antibody to investigate the precise tissue localization of FADS2, the rate-limiting enzyme for HUFA biosynthesis^14^. We discovered that in the testis, FADS2 is highly expressed in Leydig cells. We also used Leydig cells to examine the effects of disruption of this enzyme, which resulted in a drastic decrease of omega-6 HUFAs and reduced capacity to produce steroid hormones. Our findings uncovered a previously unappreciated link between omega-6 HUFAs and male steroid hormone production in the male reproductive system.

## Results

### FADS2 is highly expressed in Leydig cells

HUFAs are believed to be biosynthesized from essential fatty acids mainly in the liver, where their synthetic enzymes are highly expressed^1^. To gain a deeper understanding of HUFA synthesis, we generated an antibody specific for FADS2, the rate-limiting enzyme for HUFA synthesis, and investigated the tissue distribution of this enzyme. FADS2 uniquely catalyzes two desaturation steps indispensable for the synthesis of major HUFAs including ARA, eicosapentaenoic acid (20:5n-3; EPA), and DHA (Fig. 1a). As expected, western blotting and immunohistochemical analysis revealed a high expression of FADS2 in the mouse liver (Fig. 1b and Supplementary Fig. 1). Interestingly, these analyses also revealed that FADS2 was highly expressed in Leydig cells (labeled with an antibody against HSD3B1^15^), the cells responsible for androgen production (Fig. 1c and Supplementary Fig. 2a). In addition, FADS2 was also highly expressed in theca cells in the ovary and the adrenal gland, both of which produce steroid hormones (Supplementary Fig. 2b, c). This suggests a role of FADS2 in steroid hormone production.

### Omega-6 HUFAs are drastically reduced in FADS2^–/–^ testis

**Fig. 2:**
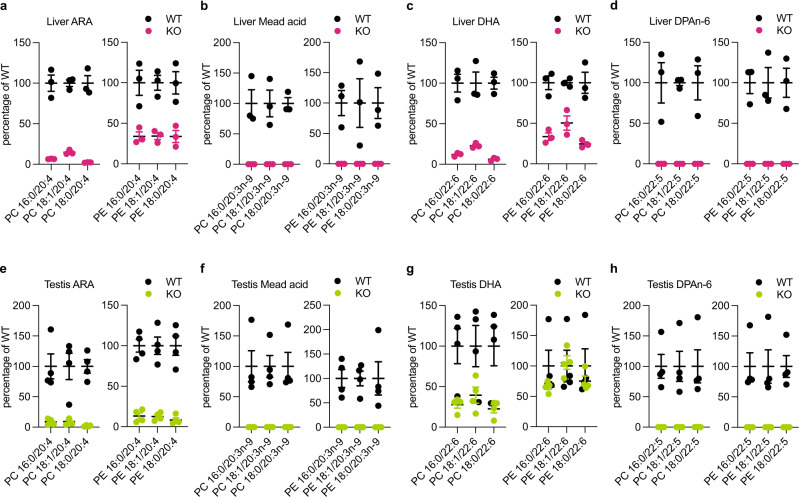
HUFAs are reduced in FADS2^−/−^ liver and testis. Quantification of HUFA species of phosphatidylcholine (PC) and phosphatidylethanolamine (PE) in livers (**a**–**d**) and testes (**e**–**h**) from FADS2^+/+^ (WT) and FADS2^−/−^ (KO) mice. The values of each molecular species are expressed as a percentage relative to WT. *n* = 3 (**a**–**d**) and *n* = 4 (**e**–**h**) for each group. Data shown are the mean ± SEM. The detailed lipidomics data are shown in Supplementary Fig. 3. ARA arachidonic acid, DHA docosahexaenoic acid, DPA n-6 docosapentaenoic acid.

Next, we performed lipidomics analysis on FADS2^−/−^ mouse tissues. To eliminate the effects of exogenously supplied HUFAs, we fed mice with a standardized rodent diet containing essential fatty acids but excluding HUFAs^5^ (see Methods). In the liver, FADS2 knockout resulted in a significant reduction of various species of HUFAs in the major phospholipids phosphatidylcholine (PC) and phosphatidylethanolamine (PE) regardless of the position of double bonds; levels of ARA- and DHA-containing PE species were less reduced in FADS2^−/−^ livers (50–75%) compared with those in FADS2^+/+^ livers (Fig. 2a–d and Supplementary Fig. 3a–f). This is consistent with the idea that HUFAs are synthesized in the liver from essential fatty acids via HUFA synthetic enzymes including FADS2. Mead acid (20:3n-9), a polyunsaturated fatty acid (PUFA), which is synthesized from oleic acid (18:1n-9) especially when HUFAs are deficient, was almost undetectable in FADS2^−/−^ liver, confirming the necessity of FADS2 in the synthesis of this PUFA (Fig. 2b)^13,16,17^. Consistent with previous studies, wild-type (WT) testes contained very high levels of omega-6 HUFAs, particularly ARA and DPAn-6 (Supplementary Fig. 3g–l). In FADS2^−/−^ testes, the levels of these omega-6 HUFAs were significantly reduced: ARA (20:4n-6) levels were reduced by more than 85% (Fig. 2e), and lipid species containing DPAn-6 were almost undetectable (Fig. 2h). In contrast, a considerable amount of DHA, especially in PE, still remained in the FADS2^−/−^ testis compared with that in the WT testis (Fig. 2g). These data indicate that the high levels of ARA and DPAn-6 in the testis, in particular, greatly relies on their production via the HUFA synthetic pathway where FADS2 plays a central role, whereas the level of DHA is sustained to some extent by another as yet unknown mechanism.

### Omega-6 HUFAs are required for steroid hormone production

**Fig. 3:**
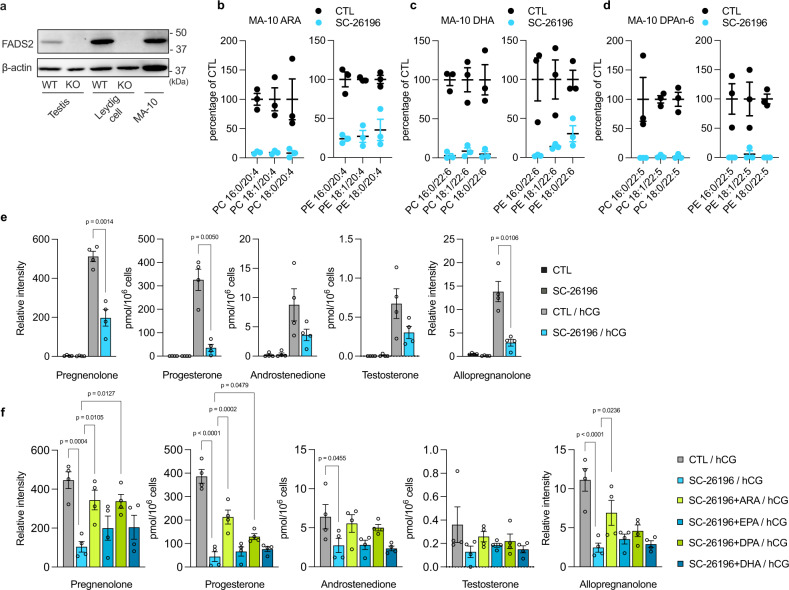
Omega-6 HUFAs are required for steroid hormone production in MA-10 cells. **a** Immunoblotting using anti-mouse FADS2 antibody of samples from testes and Leydig cells from FADS2^+/+^ (WT) and FADS2^−/−^ (KO) mice and MA-10 cells. β-actin was used as a loading control. **b**–**d** Quantification of HUFA species in MA-10 cells. The values of each molecular species were expressed as a percentage relative to CTL. *n* = 3 for each group. The detailed lipidomics data are shown in Supplementary Fig. 4. **e** Reduced steroid hormone production in MA-10 cells treated with SC-26196. Steroid hormones were extracted from the supernatant. *n* = 4 for each group. Significance is based on unpaired two-tailed *t*-test with Welch’s correction. **f** Steroid hormone production in MA-10 cells supplemented with HUFAs. Steroid hormones were extracted from the supernatant. *n* = 4 for each group. Significance is based on Dunnett’s multiple comparisons test. Data shown are the mean ± SEM. Steroid hormone levels in the cell pellets are shown in Supplementary Fig. 4f. CTL control, hCG human chorionic gonadotropin, ARA arachidonic acid, EPA eicosapentaenoic acid, DHA docosahexaenoic acid, DPA n-6 docosapentaenoic acid.

**Fig. 4:**
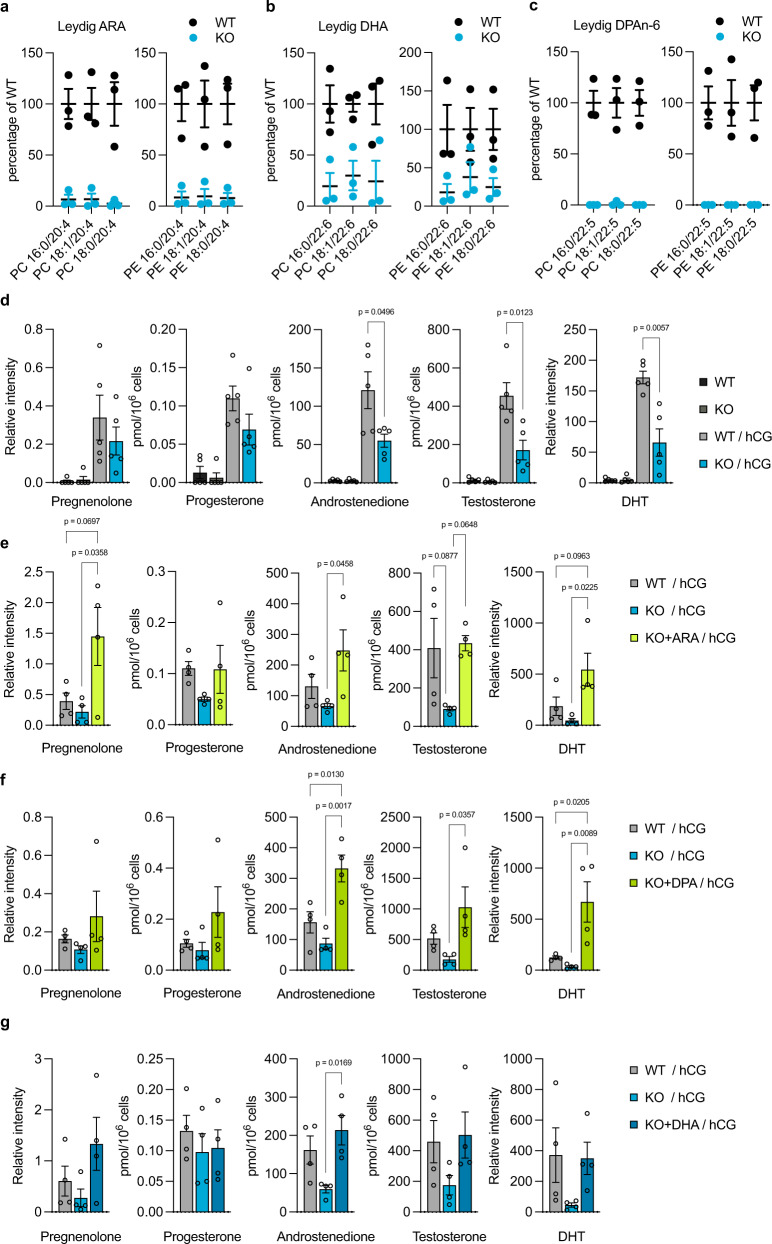
Omega-6 HUFAs are required for steroid hormone production in Leydig cells. **a**–**c** Quantification of HUFA species in Leydig cells from FADS2^+/+^ (WT) and FADS2^−/−^ (KO) mice. *n* = 3 for each group. The detailed lipidomics data are shown in Supplementary Fig. 5. **d** Reduced steroid hormone production in Leydig cells from FADS2^−/−^ mice. Steroid hormones were extracted from the supernatant. *n* = 4 for each group. Significance is based on unpaired two-tailed *t*-test with Welch’s correction. **e**–**g** Steroid hormone production in Leydig cells from FADS2^−/−^ mice supplemented with ARA (**e**), DPAn-6 (**f**), or DHA (**g**). *n* = 4 for each group. Significance is based on Tukey’s multiple comparisons test. Data shown are the mean ± SEM. Steroid hormone levels in the cell pellets were shown in Supplementary Fig. 5f–h. DHT dihydrotestosterone, hCG human chorionic gonadotropin, ARA arachidonic acid, DHA docosahexaenoic acid, DPA n-6 docosapentaenoic acid.

To investigate the role of FADS2 in Leydig cells, we first utilized the MA-10 mouse Leydig cell line, which expresses the LH receptor and produces steroid hormones upon LH stimulation. Western blotting confirmed that FADS2 was also highly expressed in MA-10 cells (Fig. 3a). Treatment with SC-26196, a selective FADS2 inhibitor^18^, resulted in the reduction of various HUFAs, suggesting that FADS2 vigorously synthesized HUFAs in this cell line (Fig. 3b–d and Supplementary Fig. 4a–e). Upon human chorionic gonadotropin (hCG) stimulation, MA-10 cells produced and released testosterone, its precursors (androstenedione, progesterone, and pregnenolone), and allopregnanolone (a progesterone metabolite) (Fig. 3e). The low level of testosterone compared with that of the precursors is due to the low expression level of CYP17A1 in this cell line^19^. In contrast, the levels of these steroid hormones were significantly reduced in SC-26196-treated cells (Fig. 3e). This reduction was rescued by supplementation with omega-6 HUFAs ARA and DPAn-6 but not with omega-3 HUFAs EPA or DHA (Fig. 3f and Supplementary Fig. 4f). To verify the role of these omega-6 HUFAs in steroidogenesis in vivo, we investigated Leydig cells isolated from testes of FADS2^+/+^ and FADS2^−/−^ mice by combined enzyme digestion and Percoll separation (see Methods). Leydig cells from FADS2^−/−^ mice showed a similar lipidomic change in the testis; the levels of omega-6 HUFAs were almost completely depleted, whereas a substantial level of DHA remained (Fig. 4a–c and Supplementary Fig. 5a–e). When incubated in the cell culture medium with hCG, FADS2^+/+^ Leydig cells produced and released testosterone as well as the steroid precursors and dihydrotestosterone (Fig. 4d). In contrast, the levels of these steroid hormones were significantly lower in FADS2^−/−^ Leydig cells (Fig. 4d). In FADS2^−/−^ Leydig cells, the mRNA expression levels of steroidogenic genes were upregulated (Supplementary Fig. 6) whereas the number of Leydig cells (per mg testis weight) was not significantly different (Supplementary Fig. 7), negating the possibility that the reduced steroid hormone production was attributed to the reduced expression of steroidogenic genes. Next, we supplemented FADS2^+/+^ and FADS2^−/−^ littermate mice with either ARA, DPAn-6, or DHA by gavage (400 mg/kg body weight, 3 times a week for 6 weeks) and tested the capacity of Leydig cells to produce steroid hormones. Interestingly, although all HUFAs exhibited a tendency toward restored steroid hormone production, DPAn-6-supplemented FADS2^−/−^ cells showed even higher steroid hormone production as compared to their wild-type littermate counterparts (Fig. 4e–g and Supplementary Fig. 5f–h). Taken together, these data indicate that omega-6 HUFAs synthesized by FADS2 in Leydig cells are necessary for the production and release of male steroid hormones.

As reported previously, FADS2^−/−^ mice exhibited a reduction in testis weight and sperm count, and the defects in sperm morphology and motility (Supplementary Fig. 8 and Supplementary Video 1, 2)^5,6,20–22^. Consistent with the reduced production of steroid hormones in isolated Leydig cells (Fig. 4d), the steroid hormone levels in serum and testes tended to be lower in FADS2^−/−^ mice (Supplementary Fig. 9a, b), whereas serum LH levels were comparable (Supplementary Fig. 9c). The weight of seminal vesicles, the development of which is highly dependent on androgens, was also lower in FADS2^−/−^ mice (Supplementary Fig. 9d, e). HUFA supplementation restored the reduction in the weight of testes and seminal vesicles (Supplementary Fig. 8a, b and Supplementary Fig. 9d, e). As reported previously, the supplementation of DHA, but not ARA, restored spermatogenesis in FADS2^−/−^ mice, as evidenced by the presence of elongated spermatids in the seminiferous tubules (Supplementary Fig. 10)^20,22^. DPAn-6 also failed to restore the defects in spermatogenesis (Supplementary Fig. 10). Taken together, these results support the idea that omega-6 HUFAs and DHA play a distinct role in testes.

### HUFA-containing cholesteryl ester species are preferred substrates for HSL

**Fig. 5:**
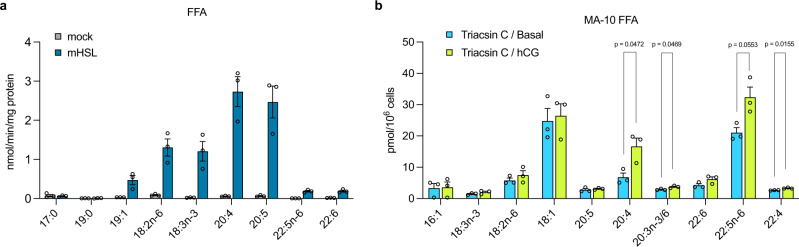
HUFA-containing cholesteryl ester species are preferred substrates for HSL. **a** The lipase activity of recombinant mouse hormone-sensitive lipase (mHSL). HEK293T cells were transfected with empty vector (mock) or mHSL-expressing vector (mHSL) and the cell lysate (1 mg/mL) was incubated with each cholesterol ester species (100 μM) for 30 min at 37 °C. FFA, free fatty acid. *n* = 3 for each group. **b** HSL-dependent FFA liberation upon human chorionic gonadotropin (hCG) stimulation in MA-10 cells. Triacsin C was pretreated to block the conversion of FFAs to fatty acyl-CoAs. The FFA levels insensitive to HSL-IN-1 were subtracted to evaluate HSL-dependent FFA production. *n* = 3 for each group. Significance is based on unpaired two-tailed *t*-test with Welch’s correction. Data shown are the mean ± SEM. The detailed data are shown in Supplementary Fig. 13.

We hypothesized that omega-6 HUFAs in cholesteryl esters are involved in the substrate preference of HSL. We first confirmed that knockout or inhibition of FADS2 drastically reduced the levels of HUFA-containing cholesteryl ester species (Supplementary Fig. 11), and that they were replenished by HUFA supplementation (Supplementary Fig 12). Next, we chemically synthesized a variety of cholesteryl ester species with different fatty acids to test the substrate specificity of HSL. Recombinant HSL transiently expressed in HEK293T cells showed robust hydrolytic activity in vitro when HUFA-containing species, such as ARA and EPA species, were used as a substrate (Fig. 5a). ARA and EPA are both C20 HUFAs with their first double bond at the ∆5 position (counted from the carboxylic acid end of the carbon chain). This structural similarity may be important for HSL’s substrate preference in vitro, as may be the case for some other lipid-metabolizing enzymes^23–25^. As we expected, recombinant HSL showed a limited capacity to hydrolyze cholesteryl esters with saturated or monounsaturated fatty acids. Unexpectedly, HSL only moderately hydrolyzed cholesteryl esters with DPAn-6 or DHA. We hypothesized that chemical properties of the substrates and/or enzyme accessibilities under these in vitro conditions may be different from those under physiological conditions (i.e., freely mixing, in vitro, vs. in or on the surface of lipid droplets of cells in tissues). To test the substrate preference of HSL in living cells, we measured the levels of free fatty acids (FFAs) liberated from cholesteryl esters upon hCG stimulation. We pretreated MA-10 cells with Triacsin C, an inhibitor of acyl-CoA synthetases, to block the conversion of FFAs to fatty acyl-CoAs. As expected, Triacsin C treatment markedly increased the levels of FFAs within the cells (Supplementary Fig. 13). We also utilized HSL-IN-1, a selective and potent inhibitor of HSL^26^, to detect HSL-dependent FFA liberation. When the cells were stimulated with hCG, the levels of ARA and DPAn-6 were markedly increased (Fig. 5b). In contrast, the levels of other FFAs such as DHA were only mildly increased (Fig. 5b). HSL-IN-1 treatment reduced the FFA levels even without hCG stimulation, showing the function of this enzyme on the basal levels (Supplementary Fig. 13). In addition, hCG stimulation did not increase FFA levels in the presence of HSL-IN-1 (Supplementary Fig. 13). Collectively, these data indicate the preference of HSL for cholesteryl esters that contain omega-6 HUFAs for the efficient liberation of cholesterol and the subsequent production of male steroid hormones.

## Discussion

In this study, we demonstrated that pharmacological or genetic inhibition of ∆6 desaturase FADS2 and concomitant depletion of HUFAs had a severe impact on male steroid hormone production in Leydig cells. We also showed that supplementation with omega-6 HUFAs such as ARA and DPAn-6 rescued the reduced capacity of steroid hormone production in FADS2-disrupted Leydig cells both in vitro and in vivo. Many studies have focused on the beneficial effects of dietary omega-3 HUFAs as these have been shown to prevent a variety of diseases including cardiovascular diseases and metabolic syndromes^27–30^. However, omega-6 HUFAs, particularly ARA, have been regarded as the major culprits for various diseases as their metabolites, such as prostaglandins and leukotrienes, elicit and aggravate inflammation. This study thus highlights a novel beneficial role of omega-6 HUFAs in the male reproductive system.

We also found that HSL, a lipase that supplies free cholesterol for steroidogenesis, preferentially hydrolyzed cholesteryl esters with omega-6 HUFAs upon hCG stimulation (Fig. 5b). Interestingly, reduction of steroid hormone production in MA-10 cells by FADS2 inhibition was only rescued by supplementation with omega-6 HUFAs (Fig. 3f and Supplementary Fig. 4f). In addition, the supplementation with omega-6 HUFAs, but not DHA, resulted in even higher steroid hormone production in FADS2^−/−^ Leydig cells than in FADS2^+/+^ cells (Fig. 4e–g and Supplementary Fig. 5f–h). HSL is also localized in adipose tissues and hydrolyzes not only cholesteryl esters but also glycerolipids, such as triacylglycerols and diacylglycerols, which are mainly composed of monounsaturated fatty acids, indicating a broad substrate specificity^31,32^. Consistently, treatment of MA-10 cells with HSL-IN-1, a selective and potent inhibitor of HSL^26^, reduced the basal levels of various FFA species including oleic acid (18:1n-9) (Supplementary Fig. 13). Therefore, the omega-6 HUFA-specific effects on steroidogenesis may not only be attributed to their chemical properties or substrate specificities. For example, ARA liberated from cholesteryl esters by HSL may be transferred to mitochondria and contribute to steroidogenesis via induction of the steroidogenic acute regulatory protein (StAR; STARD1), a protein essential for the transfer of cholesterol into mitochondria^33^. Given the residual DHA in FADS2-inhibited testes and Leydig cells (Figs. 2g, 4b and Supplementary Fig. 3, 5) and abnormally high steroid hormone levels produced in Leydig cells from FADS2^−/−^ mice supplemented with omega-6 HUFAs (Fig. 4e, f and Supplementary Fig. 5f, g), the presence of both omega-6 and omega-3 HUFAs in cholesteryl esters and/or their balance may be important for the normal production of steroid hormones. High levels of androgens have been implicated in several physiological and pathological conditions including androgenetic alopecia, acne, seborrhea, prostate cancer, and benign prostatic hypertrophy^34–37^. Interestingly, these pathological conditions have been connected with the western diet^35,38–40^, which contains high levels of omega-6 HUFAs but lower levels of omega-3 HUFAs, whereas the beneficial effects of omega-3 HUFAs on these conditions have also been described^41–44^. Omega-3 HUFAs may suppress excessive biosynthesis of male steroid hormones by balancing the omega-6/omega-3 ratio of cholesteryl esters in Leydig cells. Further studies are needed to clarify the molecular mechanisms of how omega-6 and omega-3 HUFAs modulate male steroid hormone production.

HUFAs are believed to be synthesized from essential fatty acids (i.e., linoleic acid and α-linolenic acid) in the liver and transported to other peripheral tissues via the bloodstream. However, we found that FADS2 was very highly expressed in Leydig cells of the testis (Fig. 1). This locally expressed FADS2 seems to be somewhat specialized in producing omega-6 HUFAs, especially DPAn-6. In the liver, ARA and DHA are the most abundant HUFAs and DPAn-6 is a relatively minor HUFA, whereas in the mammalian testis, DPAn-6 is one of the most abundant HUFAs. Omega-6 HUFAs are further elongated to very-long-chain (VLC) HUFAs (e.g., C28:5n-6 and C30:5n-6), which are indispensable components of germ cell sphingolipids^45,46^. In this context, Leydig cell FADS2 may have two distinct roles: (1) omega-6 HUFA synthesis for efficient steroid hormone production and (2) storage of precursors for omega-6 VLC-HUFAs that are essential for spermatogenesis. Our lipidomics analysis demonstrated the presence of not only DPAn-6 but also longer omega-6 HUFAs in cholesteryl esters of Leydig cells and showed that these HUFAs were depleted by FADS2 inhibition (Supplementary Fig. 11). Furthermore, omega-6 VLC-HUFA-containing sphingolipid species were also depleted from FADS2^−/−^ testis (Supplementary Fig. 14). These data support the role of FADS2 in supplying omega-6 VLC-HUFAs for spermatogenesis. A remaining question is how the testis or Leydig cells enrich omega-6 HUFAs. As FADS2 catalyzes the desaturation of both omega-3 and omega-6 PUFAs, we hypothesize that there are other as yet undescribed mechanisms regarding this omega-6 preference. We are currently attempting to identify key factors that are related to this propensity to selectively synthesize or accumulate omega-6 HUFAs in Leydig cells.

Previous studies demonstrated that the development of Sertoli cells was normal but their polarity and the blood–testis barrier were disrupted in FADS2^−/−^ testis^6,22^. They suggested that HUFAs biosynthesized by FADS2 were indispensable membrane components for Sertoli cell polarity and blood–testis barrier formation. Given that steroid hormones are also important for Sertoli cell function and development, and thus for germ cell differentiation and development, the reduced steroid hormone production by FADS2 deletion might also contribute to the testicular phenotypes observed in FADS2^−/−^ mice. Further investigation would be required to delineate the influence of the reduced capacity of steroidogenesis in FADS2^−/−^ Leydig cells on testicular development and spermatogenesis.

In this study, we used hCG, instead of LH, to induce steroidogenesis in Leydig cells. LH and hCG have been widely accepted to act as an agonist of the LH receptor with equivalent potency. However, recent studies have suggested that these two ligands have different pharmacological properties^47^. For example, LH was less potent than hCG and acted as a partial agonist triggering weaker maximum β-arrestin 2 recruitment and progesterone production in a murine Leydig tumor cell line MLTC-1 cells^48^. In addition, FADS2 was also highly expressed in other steroidogenic cells and tissues such as adrenal gland, where adrenocorticotropin acts to trigger steroidogenesis. Therefore, the use of different agonists and/or cell models may be required to fully understand the role of FADS2 and its products in steroidogenesis.

In conclusion, we report an essential role of omega-6 HUFAs in male steroid hormone production. These HUFAs are vigorously generated by the ∆6 desaturase FADS2 in Leydig cells. *FADS2* gene polymorphisms have been implicated in various pathophysiological conditions^49–52^. We expect that future studies will prove the clinical significance of genetic variance of *FADS2* gene and omega-6 HUFAs in human male hormone production.

## Methods

### Animal experiments

Mice were housed under controlled temperature and humidity and under a 12:12-h dark–light cycle. FADS2^−/−^ mice were obtained by heterozygous mating. After mating, these mice were distinguished from FADS2^+/−^ and FADS2^+/+^ mice by PCR using forward (5′-GCTAGCTAGTGAAGGCGAGG-3′) and reverse (5′-TGTAGACCTTGCGGTCGATG-3′) primers. Male FADS2^−/−^ mice and littermate WT mice were weaned when 3 weeks old and fed AIN-93G diet for 8 weeks. For HUFA supplementation, mice were fed AIN-93G for 10 weeks with HUFA supplementation during the last 6 weeks, three times weekly, via gavage at a dose of 400 mg/kg body weight in saline. All animal experiments were approved by and performed in accordance with the guidelines of the Animal Research Committee of the Juntendo University Graduate School of Medicine (approval number: 2020147). All experiments involving gene recombination were approved by and performed in accordance with the guidelines of the Biosafety Committee of the Juntendo University Graduate School of Medicine (approval number: 31-4).

### Generation of anti-mFADS2 polyclonal antibody

Rabbit polyclonal anti-mouse FADS2 antibodies were raised against synthetic peptides EIQKHNLRTDRWLVIDRKV or DIVSSLKKSGELWLDAYLHK at Immuno-Biological Laboratories Co., Ltd. (Fujioka, Japan). The antibodies were affinity purified using Protein G Sepharose chromatography. The antibody to the aforementioned first peptide was used for further analyses.

### Western blotting

Cells and tissues were homogenized in lysis buffer containing 20 mM Tris–HCl (pH 7.4), 1% Triton X-100, 0.5% NP-40, 5 mM EDTA, and protease inhibitor cocktail (Nacalai Tesque 25955). The protein concentration in cell lysates was determined with the Pierce^®^ 660 nm Protein Assay Reagent (Thermo Scientific). Samples were denatured by the addition of 4 × SDS/PAGE loading buffer (240 mM Tris–HCl, pH 6.8; 40% glycerol; 8% SDS; 0.1% bromophenol blue; 20% 2-mercaptoethanol) and heated at 95 °C for 5 min. Protein samples and pre-stained molecular weight standards (Precision Plus Protein™ Dual Color Standards, Bio-Rad) were resolved on 10% SDS-polyacrylamide gels and transferred onto Immobilon^®^-P transfer membranes (Millipore Sigma, USA). The membranes were blocked using 5% skimmed milk in Tris-buffered saline, with 0.05% Tween 20 (Sigma) at room temperature for 1 h. The membranes were then incubated with primary antibodies at 4 °C for 12 h, washed three times with the wash buffer (Tris-buffered saline with 0.05% Tween-20) for 5 min each, and then incubated with secondary antibodies at room temperature for 2 h. The primary antibodies used were anti-mFADS2 antibody (0.39 mg/ml, 1:300, rabbit, generated in this study), anti-mHSD3B1 antibody (1.72 mg/ml, 1:1000, rat, a Leydig cell marker), anti-GATA1 antibody (1:100, rat, N6, Santa Cruz Biotechnology, a Sertoli cell marker), anti-DDX4 antibody (1:1000, rabbit, 51042-1-AP, Proteintech, a germ cell marker), anti-GAPDH antibody (1:500, mouse, 6C5, Santa Cruz Biotechnology), and anti-β-actin (1:200, mouse, AC-15, Santa Cruz Biotechnology). The secondary antibodies used were anti-rabbit IgG antibody, anti-rat IgG antibody, or anti-mouse IgG antibody conjugated to horseradish peroxidase (GE Healthcare). The membranes were washed with the wash buffer three times for 5 min each and developed with ECL reagents (GE Healthcare). Immunoreactive proteins were visualized using ImageQuant LAS 4000 mini (GE Healthcare, USA).

### Immunohistochemistry

To prepare tissue sections, tissues were extracted and frozen in Tissue-Tek^®^ OCT compound with liquid nitrogen. The sections were fixed for 10 min with cold acetone on shaker. After dissolving the compound using water, tissue sections were incubated with blocking buffer (10% normal goat serum and 1% bovine serum albumin) for 20 min and then incubated with avidin and biotin blocking solutions (Vector Laboratories, Inc., USA) for 15 min at room temperature. For liver, ovary, and adrenal gland sections, anti-mFADS2 antibody (0.39 mg/ml, 1:100, rabbit, generated in this study) diluted in PBS with 1% BSA and 0.1% Triton X-100 was added and incubated overnight at 4 °C. After washing with PBS with 0.05% Tween 20 three times, the tissue sections were incubated with biotinylated anti-rabbit IgG secondary antibody (1:300, Vector Laboratories, Inc., USA) for 30 min at room temperature. After washing three times with PBS with 0.05% Tween 20, the sections were incubated with streptavidin-Alexa Fluor 488 (1:300, Vector Laboratories, Inc.) for 30 min at room temperature. For testis sections, anti-mFADS2 antibody (0.39 mg/ml, 1:50, rabbit, generated in this study) and anti-mHSD3B1 antibody (1.72 mg/ml, 1:50, rat) diluted in PBS with 1% BSA and 0.1% Triton X-100 was added and incubated overnight at 4 °C. After washing with PBS with 0.05% Tween 20 three times, the tissue sections were incubated with biotinylated anti-rat IgG secondary antibody (1:300, Vector Laboratories, Inc., USA) for 30 min at room temperature. After washing three times with PBS with 0.05% Tween 20, the sections were incubated with Alexa Fluor 488 goat anti-rabbit IgG secondary antibody (1:300, Invitrogen, Inc., USA) and streptavidin-Alexa Fluor 594 (1:300, Invitrogen, Inc., USA) for 30 min at room temperature. The sections were sealed with Antifade Mounting Medium with 4′,6-diamidino-2-phenylindole dihydrochloride (DAPI; Vector Laboratories, Inc.). Images were obtained using ApoTome.2 fluorescent microscope (Zeiss, Germany).

### Mass spectrometry analysis for phospholipids, cholesteryl esters, and sphingolipids

Lipids were extracted using the method of Bligh and Dyer with internal standards. The organic (lower) phase was transferred to a clean vial and dried under a nitrogen stream. The lipids were resolubilized in methanol (MeOH) and stored at −80 °C. A portion of the extracted lipids was injected onto an ultra-high-performance liquid chromatography–electrospray ionization (ESI)–tandem mass spectrometry system. LC separation was performed on an ACQUITY UPLC™ BEH C18 column (1.7 µm, 2.1  ×  100 mm; Waters, Milford, MA, USA) coupled to an ACQUITY UPLC™ BEH C18 VanGuard™ Pre-column (1.7 µm, 2.1   ×  5 mm; Waters). Mobile phase A was 60:40 (v/v%) acetonitrile/H_2_O containing10 mM ammonium formate and 0.1% (v/v) formic acid, and mobile phase B was 90:10 (v/v%) isopropanol/acetonitrile containing 10 mM ammonium formate and 0.1% (v/v) formic acid. The LC gradient consisted of 20% B for 2 min, a linear gradient to 60% B over 4 min, a linear gradient to 100% B over 16 min, and equilibration with 20% B for 5 min (27 min total run time). The flow rate was 0.3 mL/min, and the column temperature was 55 °C. Multiple reaction monitoring (MRM) was performed using a Xevo™ TQ-S micro triple quadrupole mass spectrometry system (Waters) equipped with an ESI source. The ESI capillary voltage was set at 1.0 kV, and the sampling cone was set at 30 V. The source temperature was 150 °C, desolvation temperature was 500 °C, and desolvation gas flow was 1000 L/h. The cone gas flow was 50 L/h.

### Cell culture and treatment

HEK293T cells (from the American Type Culture Collection) were maintained in Dulbecco’s Modified Eagle’s medium (DMEM) containing 10% fetal bovine serum (FBS) and penicillin/streptomycin at 37 °C in a humidified atmosphere of 5% CO_2_. MA-10 cells (kindly provided by Dr. Ken-Ichirou Morohashi’s lab at Department of Molecular Biology, Graduate School of Medical Sciences, Kyushu University, Fukuoka, Japan) were maintained in Waymouth’s medium supplemented with 15% horse serum and penicillin/streptomycin at 37 °C in a humidified atmosphere of 5% CO_2_. MA-10 cells were seeded at 4.0 × 10^5^ cells/well in 6-well plates in the presence of 10 μM SC-26196 (inhibitor of FADS2; Cayman) or dimethyl sulfoxide (DMSO) with/without 20 μM 20:4n-6, 20:5n-3, 22:5n-6, or 22:6n-3 (NuChek Prep, Inc., Elysian, MN) for 72 h. Cells were washed with PBS and then stimulated with serum-free medium containing 20 U/mL hCG (ASKA Animal Health Co., Ltd., Japan) for 2 h. The supernatants and cells were then harvested with MeOH:H_2_O (2/0.7, v/v) for lipid analysis. HSL-IN-1 was purchased from MedchemExpress LLC., Monmouth Junction, USA.

### Isolation of Leydig cells

Leydig cells were isolated from FADS2^+/+^ and FADS2^−/−^ testes by combined enzyme digestion and Percoll separation, as described previously with a slight modification^53^. Briefly, seminiferous tubules were placed into 10 mL of enzymatic solution containing 1 mg/mL collagenase type I (FUJIFILM, Japan), 1 mg/mL hyaluronidase (Tokyo Chemical industry Co., Ltd., Japan), and 50 µg/mL DNase I in 25 mM HEPES buffer (pH 7.4). The seminiferous tubules were digested for 20 min at 34 °C in a shaker. The dispersed cells in the enzymatic solution were mixed with 20 mL of EBSS-BSA (0.7 mg/mL) and placed on ice for 5 min. The supernatant was filtered through a 70 μm cell strainer and centrifuged at 280 × *g* for 5 min. After centrifugation, the supernatant was discarded, and 5 mL of EBSS-BSA (2.5 mg/mL) was added. The cell solution was superposed on a Percoll layer (65%, 25%, and 12%) and centrifuged at 860 × *g* for 20 min. Afterward, the cell fraction between the 65% and 25% Percoll layers was collected and then 20 mL of EBSS-BSA (2.5 mg/mL) was added and centrifuged at 280 × *g* for 5 min at 4 °C. Isolated Leydig cells were counted and incubated in serum-free Waymouth’s medium supplemented with penicillin/streptomycin containing 20 U/mL of hCG for 2 h at 37 °C in a humidified atmosphere of 5% CO_2_. The supernatants and cells were then harvested with MeOH:H_2_O (2/0.7, v/v) for lipid analysis.

### Steroid derivatization

Pregnenolone, progesterone, androstenedione, testosterone, allopregnanolone, and corticosterone were derivatized as described previously with a slight modification^54^. Lipid extracts (50 µL) were dissolved in MeOH and were derivatized with 50 µL of Girard’s reagent T (5 mg/mL in MeOH containing 0.1% trifluoroacetic acid) by heating at 40 °C for 60 min; the reaction was stopped with 10 µL of water. Dihydrotestosterone was derivatized as described previously with a slight modification^55^. Lipid extracts (50 µL) dissolved in MeOH were dried under a nitrogen stream, reacted with 60 μL of 2-fluoro-1-methylpyridinium *p*-toluenesulfonate (10 mg dissolved in 0.15 mL of acetonitrile, 0.5 mL of dichloromethane, and 6 μL of triethylamine), and incubated at room temperature for 1 h.

### Mass spectrometry analysis for steroid hormones

Intracellular and extracellular steroid hormones were measured from cell pellets and culture supernatants, respectively. A 1-mL aliquot of the supernatant from MA-10 and Leydig cell culture was collected, and 2.5 mL MeOH was added; cells were collected in a ratio of 2/0.7 (v/v) MeOH/H_2_O, and lipids were extracted as described above. LC separation was performed using the same column components and dimensions as described earlier. Mobile phase A was H_2_O containing 0.1% (v/v) formic acid, and mobile phase B was acetonitrile containing 0.1% (v/v) formic acid. LC gradient consisted of 95% A for the initial stage, a linear gradient to 95% B over 18 min, and equilibration with 95% A for 12 min (30 min total run time). The flow rate was 0.2 mL/min, and the column temperature was 55 °C. MRM was performed with the positive ion mode using the same conditions described above. Testosterone (2,3,4-13C3, 99%) (Cambridge Isotope Laboratories, Inc., USA) was used as an internal standard.

### Synthesis of cholesteryl esters

Cholesteryl esters were synthesized from cholesterol and fatty acids (16:0, 17:0, 19:0, 18:2n-6, 18:3n-3, 20:4n-6, 20:5n-3, 22:5n-6, and 22:6n-3; NuChek Prep, Inc., Elysian, MN) as described previously with a slight modification^56^. In brief, each fatty acid (10 mg) was dissolved in 0.5 mL of oxalyl chloride and heated at 50 °C for 24 h. The excess reagent was removed under a nitrogen stream, and a solution of cholesterol (30 mg) and pyridine (100 µL) in dry toluene (2 mL) was added. The mixture was kept at 50 °C overnight, and the excess solvent and reagents were again evaporated off. The final product was separated and purified using one-dimensional preparative thin-layer chromatography on silica gel 60 F_254_ plates (Merck, Darmstadt, Germany) in hexane:diethyl ether:acetic acid (70:30:1, v/v%).

### Plasmid

Full-length mouse HSL (mHSL) was amplified using PCR from mouse testis cDNA with primers mHsl forward (5′-AAATAAAGCTTATGGAGCCGGCCGTGGAATC-3′) and mHsl reverse (5′-ATTTTTCTAGAGTTCAGTGGTGCAGCAGGCG-3′) and was cloned into the pcDNA3.1/myc-His vector with a C-terminal myc-His tag via HindIII and XbaI.

### In vitro enzyme assay

Detection of cholesteryl ester hydrolysis was accomplished via mass spectrometric detection of the release of fatty acid from substrates. HEK293T cells were seeded at 5.0 × 10^5^ cells/well in 6-well plates in DMEM containing 1% FBS. After 24 h, the cells were transfected with mHSL-myc-His vector using polyethylenimine Max (Polysciences). 48 h after transfection, the cells were washed with PBS, collected, and the cell pellet was stored at −80 °C until use. The cell pellets were homogenized with a probe sonicator in PBS and the lysate was diluted to 1 mg/ml for enzyme assay. The cell lysate (1 mg/mL in 100 µL) was incubated with each cholesterol ester (100 µM) at 37 °C for 30 min. The reaction was stopped with 1 mL of MeOH and 300 μL of H_2_O. Lipids were extracted using the method of Bligh and Dyer with an internal standard. FFAs were quantified using LC-MS as described previously^57^. The extracted lipids were resolubilized in MeOH containing 0.1% (v/v) formic acid. LC separation was performed on an ACQUITY UPLC™ BEH C18 column (1.7 µm, 2.1 mm  ×  50 mm; Waters, Milford, MA, USA) coupled to an ACQUITY UPLC™ BEH C18 VanGuard™ Pre-column (1.7 µm, 2.1 mm ×  5 mm; Waters). Mobile phase A was H_2_O/acetonitrile = 80/20 (v/v%), and mobile phase B was isopropanol/acetonitrile = 80/20 (v/v%). The LC method consisted of 70% B for 15 min, a linear gradient to 100% B over 2 min, a linear gradient to 70% B over 8 min, and equilibration with 70% B for 5 min (30 min total run time). The flow rate was 0.3 mL/min, and the column temperature was 55 °C. The eluent was combined with a post-column infusion of 0.1% (v/v) NH_4_OH at an infusion flow rate of 5 µL/min. Pseudo-MRM was performed using a Xevo™ TQ-S micro triple quadrupole mass spectrometry system (Waters) equipped with an ESI source. The ESI capillary voltage was set at 1.0 kV, and the sampling cone was set at 30 V. The source temperature was set at 150 °C, desolvation temperature was set at 500 °C, and desolvation gas flow was 1000 L/h. The cone gas flow was 50 L/h. The collision energy was set to 6 eV.

### FFA measurement in MA-10 cells

MA-10 cells were seeded at 4.0 × 10^5^ cells/well in 6-well plates in the presence of 10 μM DPAn-6 for 72 h. The medium was replaced with serum-free medium, and the cells were pretreated with DMSO or 5 µM Triacsin C (Cayman Chemicals, Ann Arbor, MI) for 30 min. Cells were then treated with DMSO or 10 µM HSL-IN-1 (MedchemExpress LLC., Monmouth Junction, USA) for 30 min, stimulated with 20 U/mL hCG for 1 h, and then harvested for lipid analysis.

### Statistics and reproducibility

All data were analyzed using GraphPad Prism 7 software and the results were expressed as the mean ± standard error (SEM). Significance is based on unpaired two-tailed *t*-test with Welch’s correction, Tukey’s multiple comparisons test, or Dunnett’s multiple comparisons test. Differences were considered statistically significant when *p* values were less than 0.05.

### Reporting summary

Further information on research design is available in the Nature Research Reporting Summary linked to this article.

## Supplementary information

Supplementary Information

Description of Additional Supplementary Files

Supplementary Data 1

Supplementary Video 1

Supplementary Video 2

Reporting Summary

## Data Availability

All data generated or analyzed during this study are included in this article and the supplementary information. Raw data for graphs in Figs. 2–5 are available in Supplementary Data 1. Uncropped and unedited western blot images are shown in Supplementary Fig. 15.
