# Supplementary material for: Omega-6 highly unsaturated fatty acids in Leydig cells facilitate male sex hormone production
Source: Commun Biol. 2022 Sep 21;5:1001. doi: 10.1038/s42003-022-03972-y (PMC9492697; doi:10.1038/s42003-022-03972-y)
Supplement: Supplementary file 1 — Supplementary Information [file 42003_2022_3972_MOESM1_ESM.pdf]

Supplementary Information for

"Omega-6 highly unsaturated fatty acids in Leydig cells facilitate male sex hormone production"

Keiken Ri[1], Hyeon-Cheol Lee-Okada[1, *], Takehiko Yokomizo[1]

[1]Department of Biochemistry, Juntendo University Graduate School of Medicine, Tokyo, Japan
*Correspondence should be addressed to H-C.L-O. (h-lee@juntendo.ac.jp).

This PDF file includes:

Supplementary Methods
Supplementary Figures 1 to 15
Supplementary References

Supplementary Methods

Isolation of Sertoli cells and germ cells

After Leydig cell isolation, the remaining seminiferous tubules were further digested in 10 mL of enzymatic solution containing 1 mg/mL trypsin and 50 µg/mL DNase I in DMEM for 20 min at 34°C in a shaker. After addition of 2 mL of FBS, the cells were filtered through a 100 µm cell strainer and centrifuged at 50 × *g* for 5 min to separate the pellet containing Sertoli cells and germ cells of higher density and the supernatant containing lighter testicular germ cells and the majority of heads and tails of testicular sperm and elongated spermatids[1]. The supernatant was washed 3 times by centrifugation (800 × *g* for 3 min) in PBS to collect germ cells. The pelleted cells containing Sertoli cells were seeded onto 10-cm dishes and cultured in DMEM containing 10% FBS and penicillin/streptomycin at 37°C in a humidified atmosphere of 5% CO_2. The next day, floating germ cells were collected and Sertoli cells attached to the dish were further cultured for 6 days to remove the remaining dead germ cells[2].

LH measurement

Serum LH levels were measured using the Rodent LH ELISA test kit (Endocrine Technologies Inc., Newark, CA, USA) according to the manufacturer's instructions.

Real-Time Reverse Transcriptase PCR

Total RNA was isolated from cells using RNeasy® Plus Mini Kit (Qiagen) according to the manufacturer's instructions. Purified RNA was reverse transcribed into cDNA using SuperScript III Kit (Invitrogen). Semiquantitative real-time PCR was performed using SYBR® Green PCR Master Mix (Applied Biosystems) on a LightCycler 96 (Roche). 18S

rRNA was used as a reference gene. The sequences of the primers used were as follows: *Cyp11a1* forward 5'-CACAGACGCATCAAGCAGCAAAA-3' and reverse 5'-GCATTGATGAACCGCTGGGC-3', *Cyp17a1* forward 5'-GCTGGTATTCAGCACCTTTTCC-3' and reverse 5'-GTTGGCTTCCTGACATATCATCTTC-3', *Cyp19a1* forward 5'-CGGGCTACGTGGATGTGTT-3' and reverse 5'-GAGCTTGCCAGGCGTTAAAG-3', *Hsd3b1* forward 5'-GTTTGTGGGCCAGAGGATCA-3' and reverse 5'-GGTCTTTGTCTGCAGCTTGGA-3', *Hsd3b6* forward 5'-CCCAGGCAGACCATCCTAGA-3' and reverse 5'-TTGCCCGTACAACCGAGAA-3', *Hsd17b3* forward 5'-TCAATGGGACAATGGGCAGT-3' and reverse 5'-GCTGTGTCATCTTGACTACG-3', *Hsd17b7* forward 5'-CCACCTGTGTTTGGCGTGTA-3' and reverse 5'-GAGGTTGAATTGTGGATTAGGCA-3', and *18S rRNA* forward 5'-GTAACCCGTTGAACCCCATT-3' and reverse 5'-CCATCCAATCGGTAGTAGCG-3'.

Sperm motility

Sperm motility was monitored using an IVOS II instrument (Hamilton Thorne).

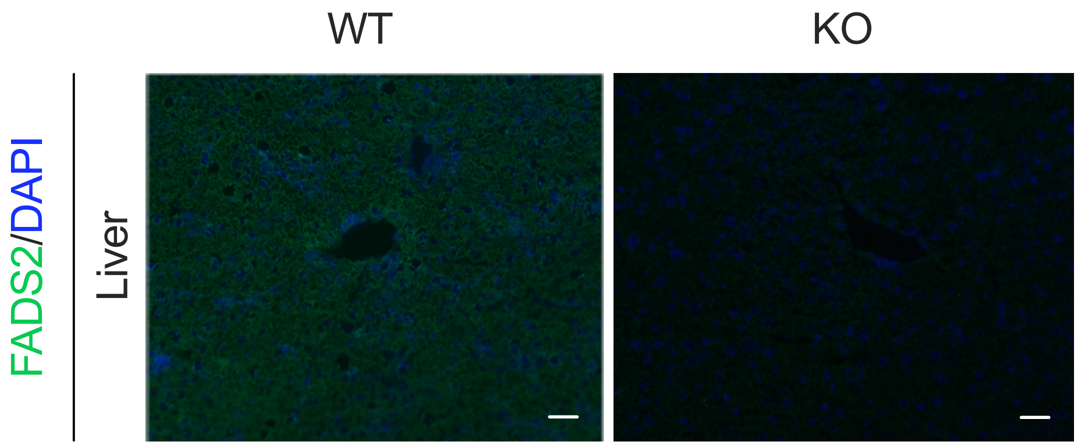

Supplementary Figure 1 FADS2 is highly expressed in the liver.

Immunohistochemical analysis of liver sections from 12-week-old $FADS2^{+/+}$ and $FADS2^{-/-}$ mice. Green, FADS2; blue, DAPI. Scale bar, 50 μm.

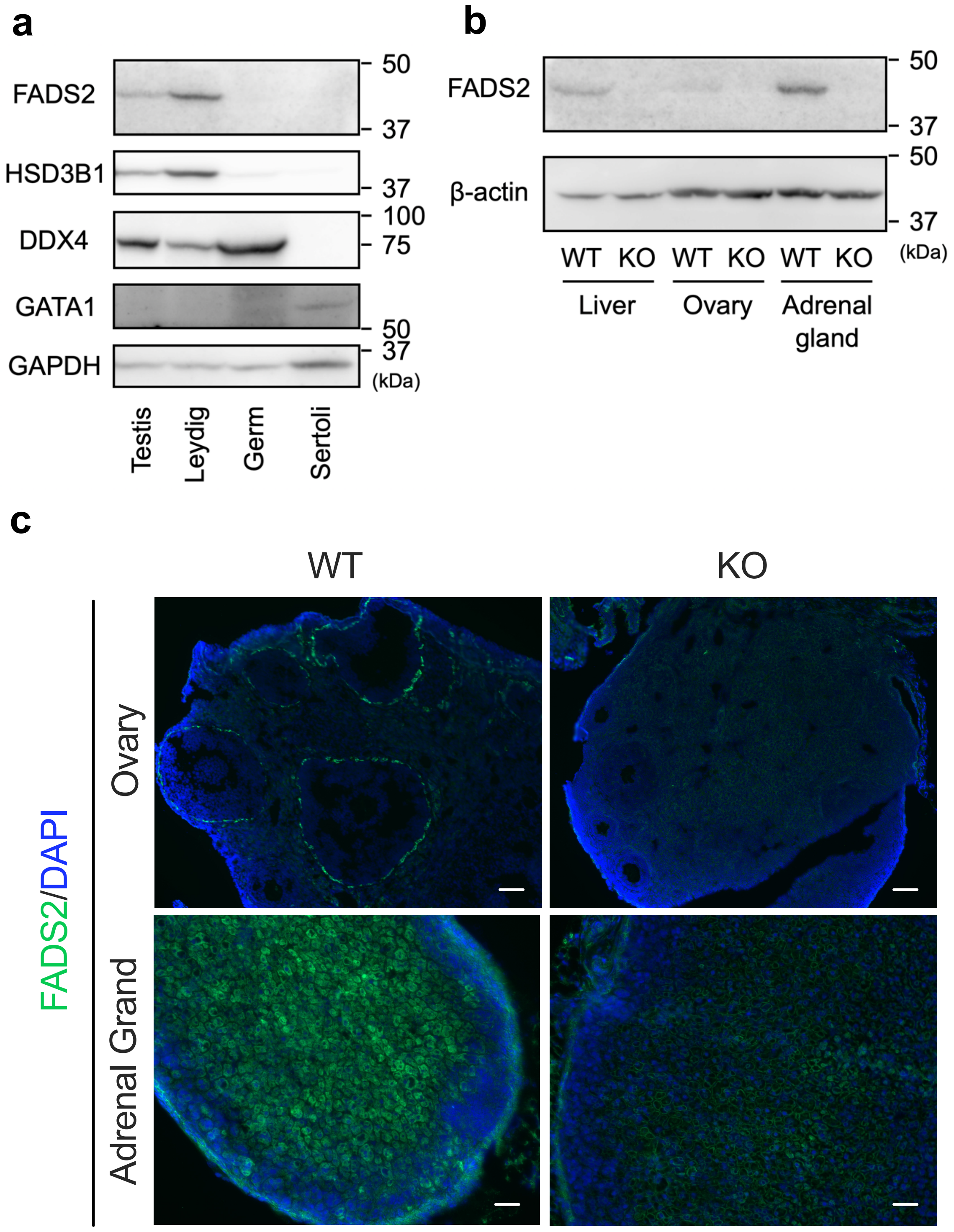

Supplementary Figure 2 Expression of FADS2 in steroidogenic cells and tissues.
a Immunoblot analysis of FADS2 in Leydig cells, germ cells, and Sertoli cells isolated from testes. GAPDH was used as a loading control. **b** Immunoblot analysis of FADS2 in the liver, ovary, and adrenal gland from FADS2$^{+/+}$ (WT) and FADS2$^{-/-}$ (KO) female mice. β-actin was used as a loading control. **c** Immunohistochemical analysis of ovary and adrenal gland sections from 12-week-old FADS2$^{+/+}$ and FADS2$^{-/-}$ mice. Green, FADS2; blue, DAPI. Scale bar, 50 μm.

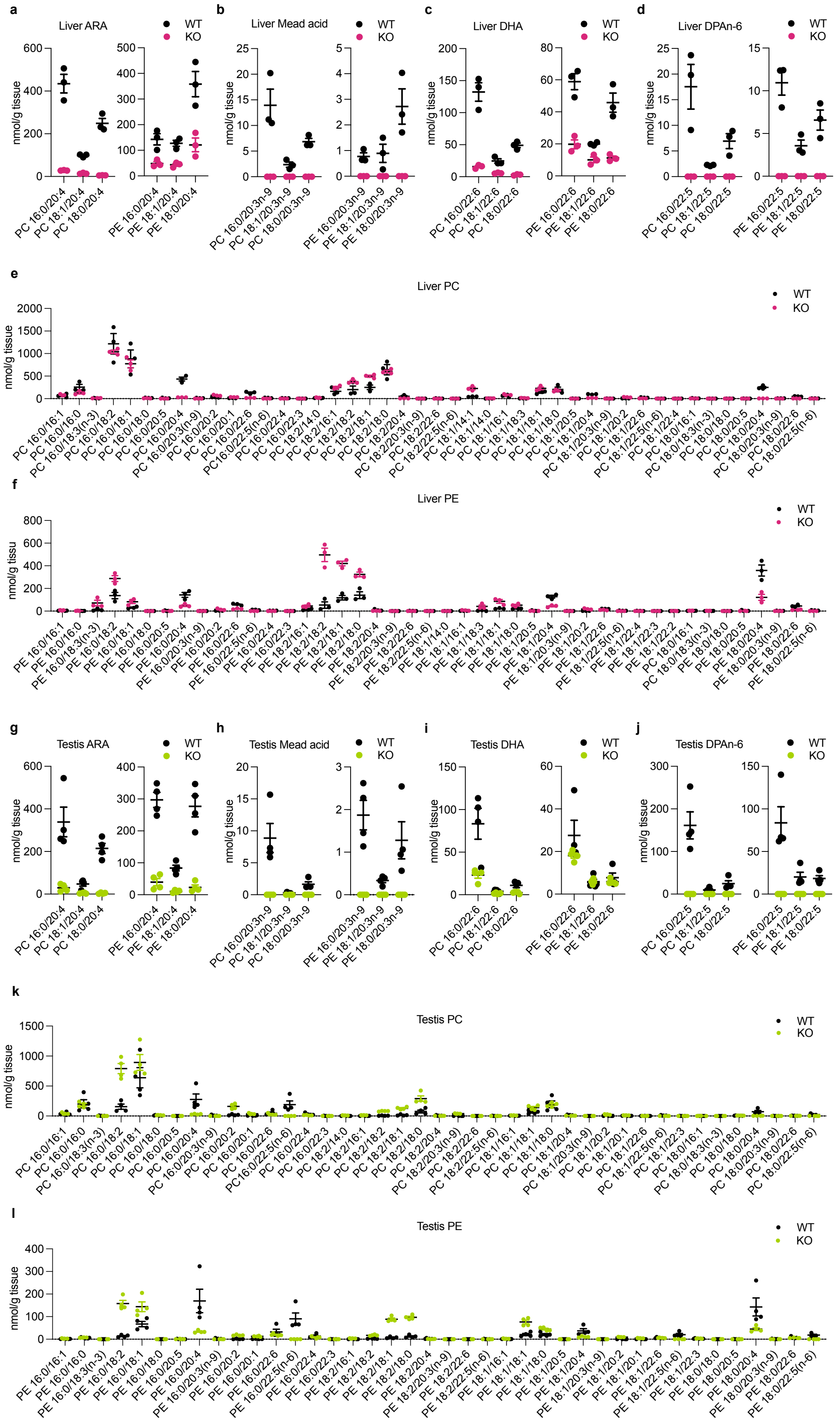

Supplementary Figure 3 Reduction of HUFAs in FADS2$^{-/-}$ livers and testes. Quantification of fatty acid molecular species of PC and PE in livers (**a**–**f**) and testes (**g**–**l**) from FADS2$^{+/+}$ (WT) and FADS2$^{-/-}$ (KO) mice. n = 3 (**a**–**f**) and n = 4 (**g**–**l**) for each group. Data shown are the mean ± SEM.

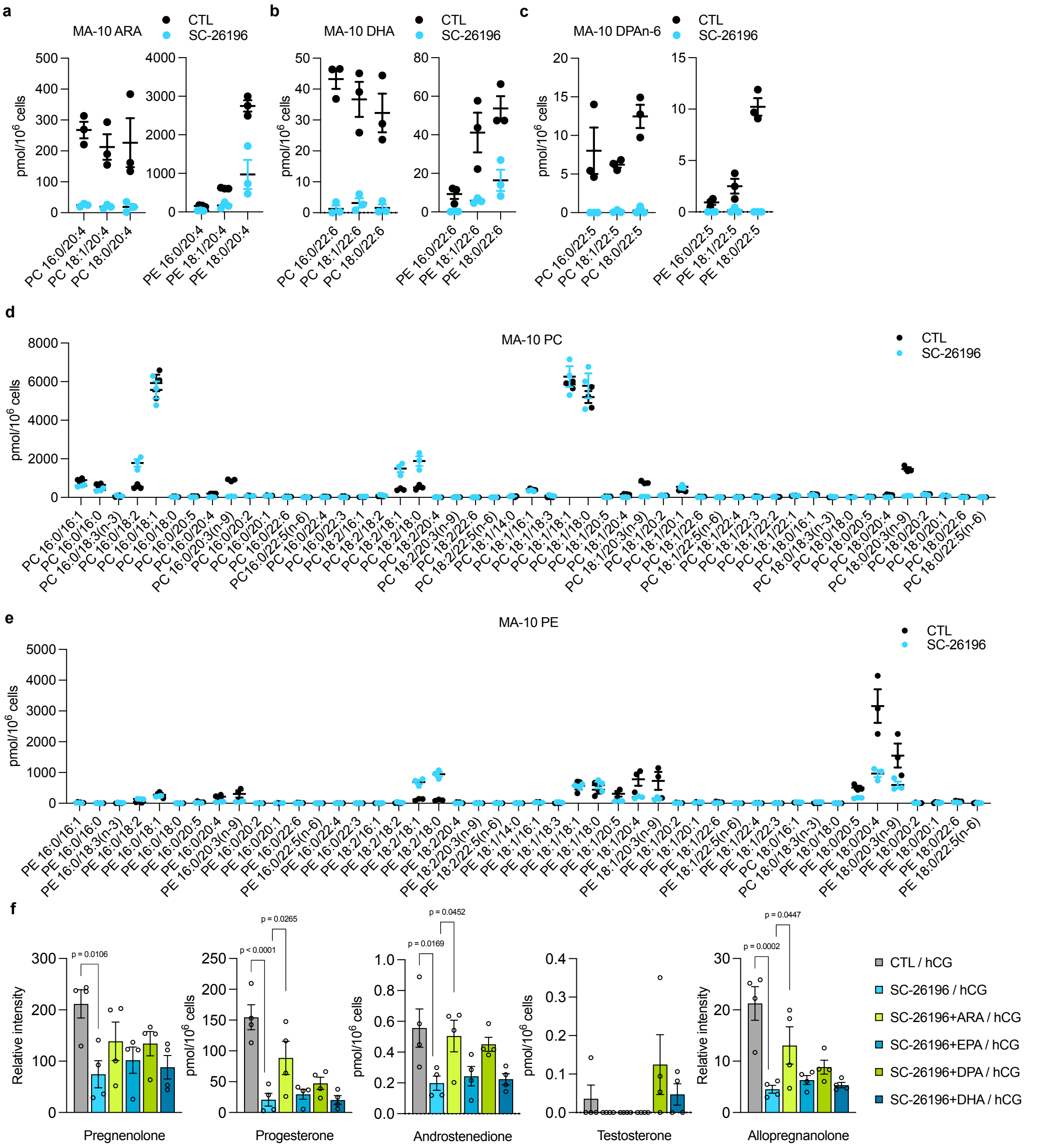

Supplementary Figure 4 Omega-6 HUFAs are required for steroid hormone production in MA-10 cells.

a–e Quantification of fatty acid molecular species of PC and PE in MA-10 cells. n = 3 for each group. **f** Steroid hormone production in MA-10 cells supplemented with HUFAs. n = 4 for each group. Significance is based on Dunnett's multiple comparisons test. Data shown are the mean ± SEM. CTL, control.

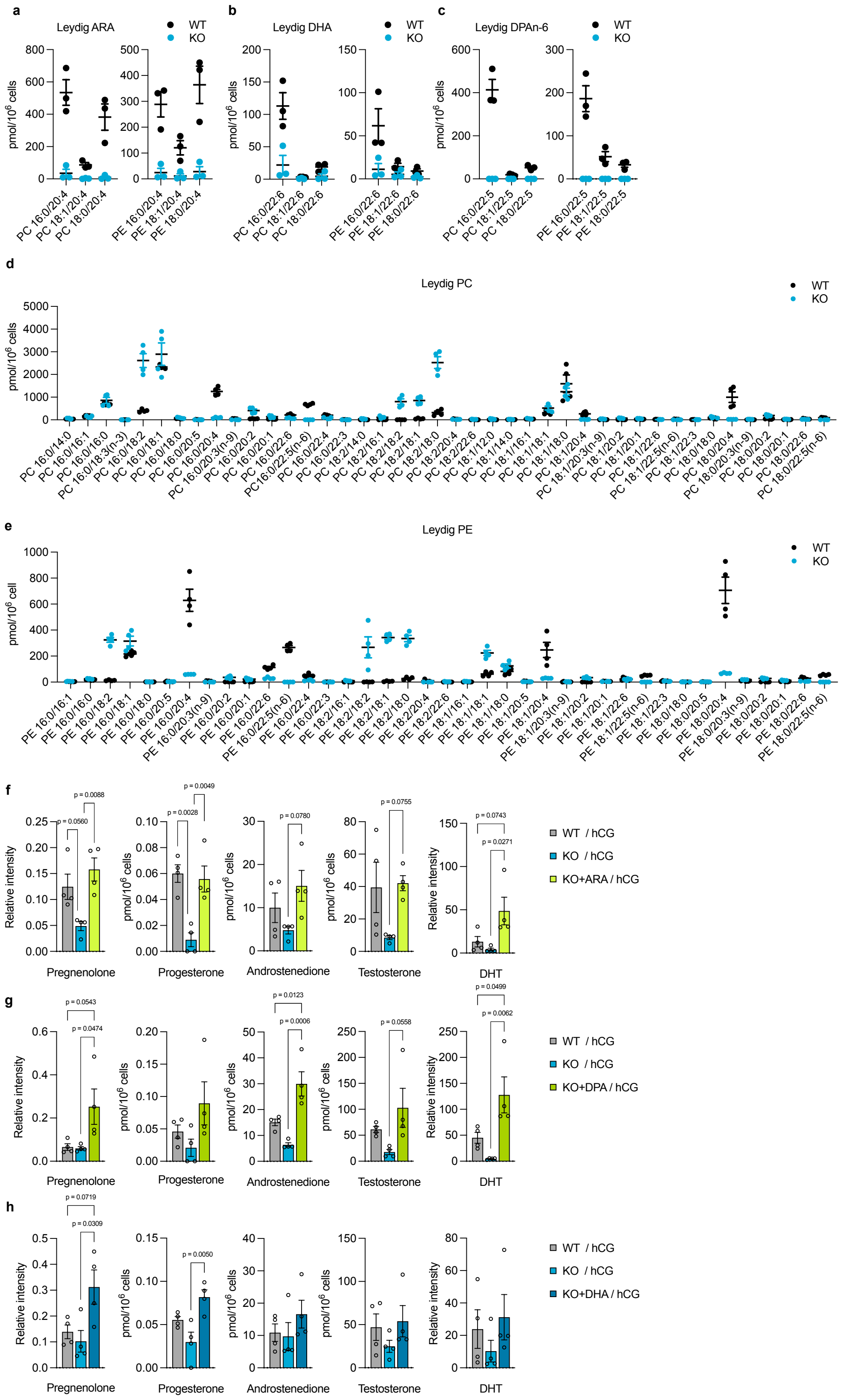

Supplementary Figure 5 Omega-6 HUFAs are required for steroid hormone production in Leydig cells.

a–e Quantification of fatty acid molecular species of PC and PE in Leydig cells from FADS2$^{+/+}$ (WT) and FADS2$^{-/-}$ (KO) mice. n = 3 or 4 for each group. **f–h** Steroid hormone production in Leydig cells from FADS2$^{+/+}$, FADS2$^{-/-}$ mice, and FADS2$^{-/-}$ mice supplemented with ARA (**f**), DPAn-6 (**g**), or DHA (**h**). n = 4 for each group. Significance is based on Tukey's multiple comparisons test. Data shown are the mean ± SEM. DHT, dihydrotestosterone.

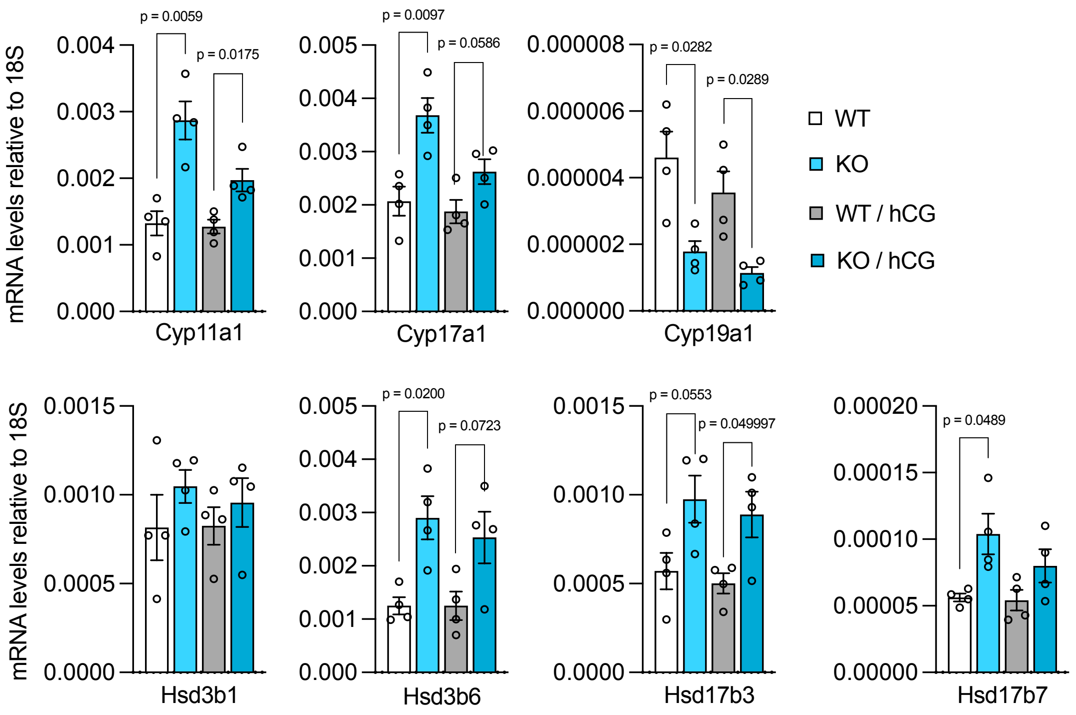

Supplementary Figure 6 mRNA expression levels of steroidogenic genes.

mRNA expression levels of genes necessary for testosterone synthesis were upregulated in Leydig cells from FADS2$^{-/-}$ (KO) mice as compared to FADS2$^{+/+}$ (WT) Leydig cells, whereas that of *Cyp19a1*, the gene encoding the enzyme that converts testosterone to estradiol, was downregulated in FADS2$^{-/-}$ Leydig cells. 18S rRNA was used as the reference gene. n = 4 for each group. Significance is based on unpaired two-tailed *t*-test with Welch's correction. Data shown are the mean ± SEM.

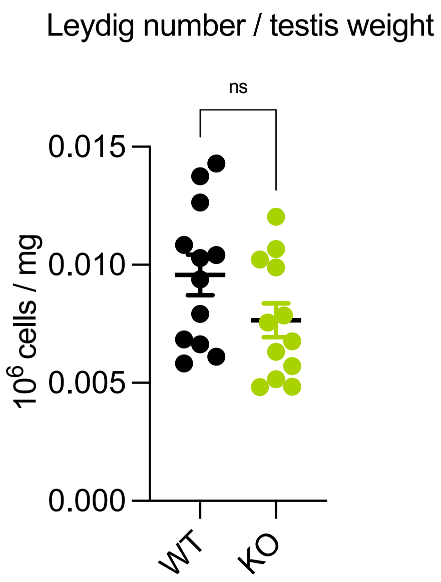

Supplementary Figure 7 The number of Leydig cells.

The number of isolated Leydig cells (per mg testis weight) was not significantly different in FADS2$^{-/-}$ (KO) mice as compared to FADS2$^{+/+}$ (WT) mice. n = 12 for each group. Significance is based on unpaired two-tailed *t*-test with Welch's correction. Data shown are the mean ± SEM. ns, not significant.

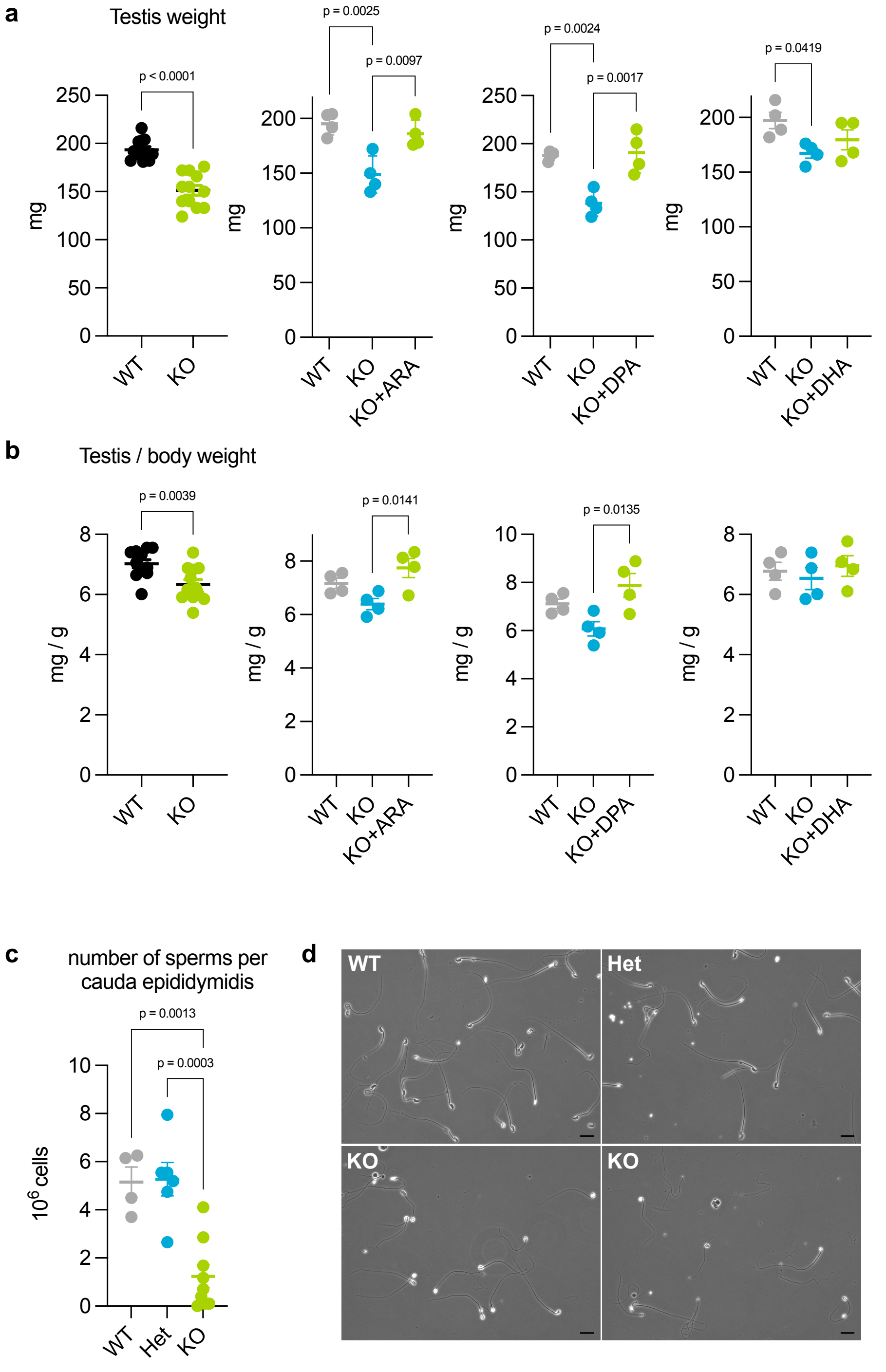

Supplementary Figure 8 Testis weight, sperm count, and sperm morphology.

a, b The testicular weight [expressed as combined weight of two testes (**a**) or its ratio to the body weight (**b**)] from FADS2$^{+/+}$ (WT) vs. FADS2$^{-/-}$ (KO) mice (left; n = 12 for each group), and FADS2$^{+/+}$ vs. FADS2$^{-/-}$ vs. FADS2$^{-/-}$ mice supplemented with ARA, DPAn-6, or DHA (right; n = 4 for each group). Significance is based on unpaired two-tailed *t*-test with Welch's correction (left) or Tukey's multiple comparisons test (right). **c** The number of sperms per cauda epididymis in FADS2$^{+/+}$ (n = 4), FADS2$^{+/-}$ (Het) (n = 6), and FADS2$^{-/-}$ (n = 9) mice. Significance is based on Tukey's multiple comparisons test. Data shown are the mean ± SEM. **d** The morphology of sperms from FADS2$^{+/+}$, FADS2$^{+/-}$, and FADS2$^{-/-}$ mice. Scale bar, 20 μm.

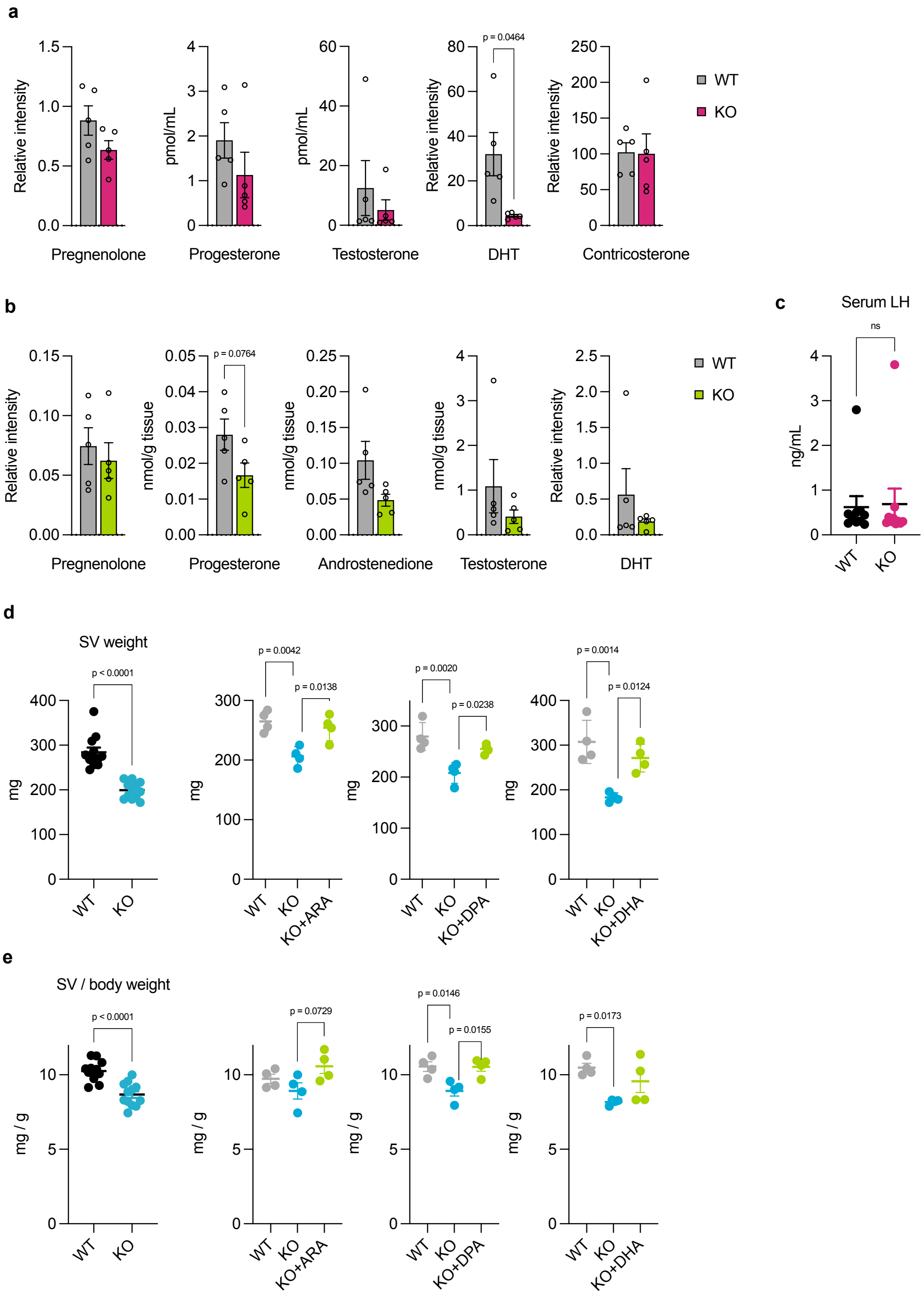

Supplementary Figure 9 Steroid hormone levels in serum and testes.

a, b Steroid hormone levels in serum (**a**) and testes (**b**) from FADS2$^{+/+}$ (WT) and FADS2$^{-/-}$ (KO) mice. n = 5 for each group. Unpaired two-tailed *t*-test with Welch's correction was performed. **c** Serum LH concentration in FADS2$^{+/+}$ and FADS2$^{-/-}$ mice. n = 10 for each group. Significance is based on unpaired two-tailed *t*-test with Welch's correction. **d, e** The weight (**d**) or weight per g body weight (**e**) of seminal vesicles (SV) from FADS2$^{+/+}$ vs. FADS2$^{-/-}$ mice (left; n = 12 for each group), and FADS2$^{+/+}$ vs. FADS2$^{-/-}$ vs. FADS2$^{-/-}$ mice supplemented with ARA, DPAn-6, or DHA (right; n = 4 for each group). Significance is based on unpaired two-tailed *t*-test with Welch's correction (left) or Tukey's multiple comparisons test (right). Data shown are the mean ± SEM. ns, not significant.

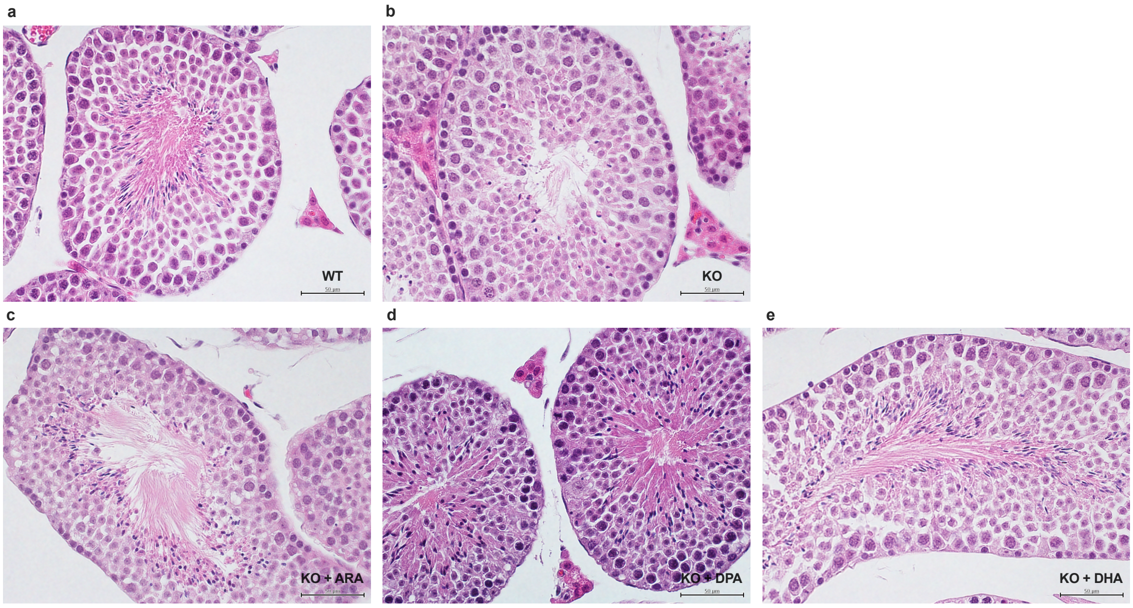

Supplementary Figure 10 Hematoxylin and eosin-stained testis sections.
Hematoxylin and eosin-stained testis sections from FADS2$^{+/+}$ (WT) (**a**), FADS2$^{-/-}$ (KO) (**b**), FADS2$^{-/-}$ mice supplemented with ARA (**c**), DPAn-6 (**d**), or DHA (**e**). Scale bar, 50 μm.

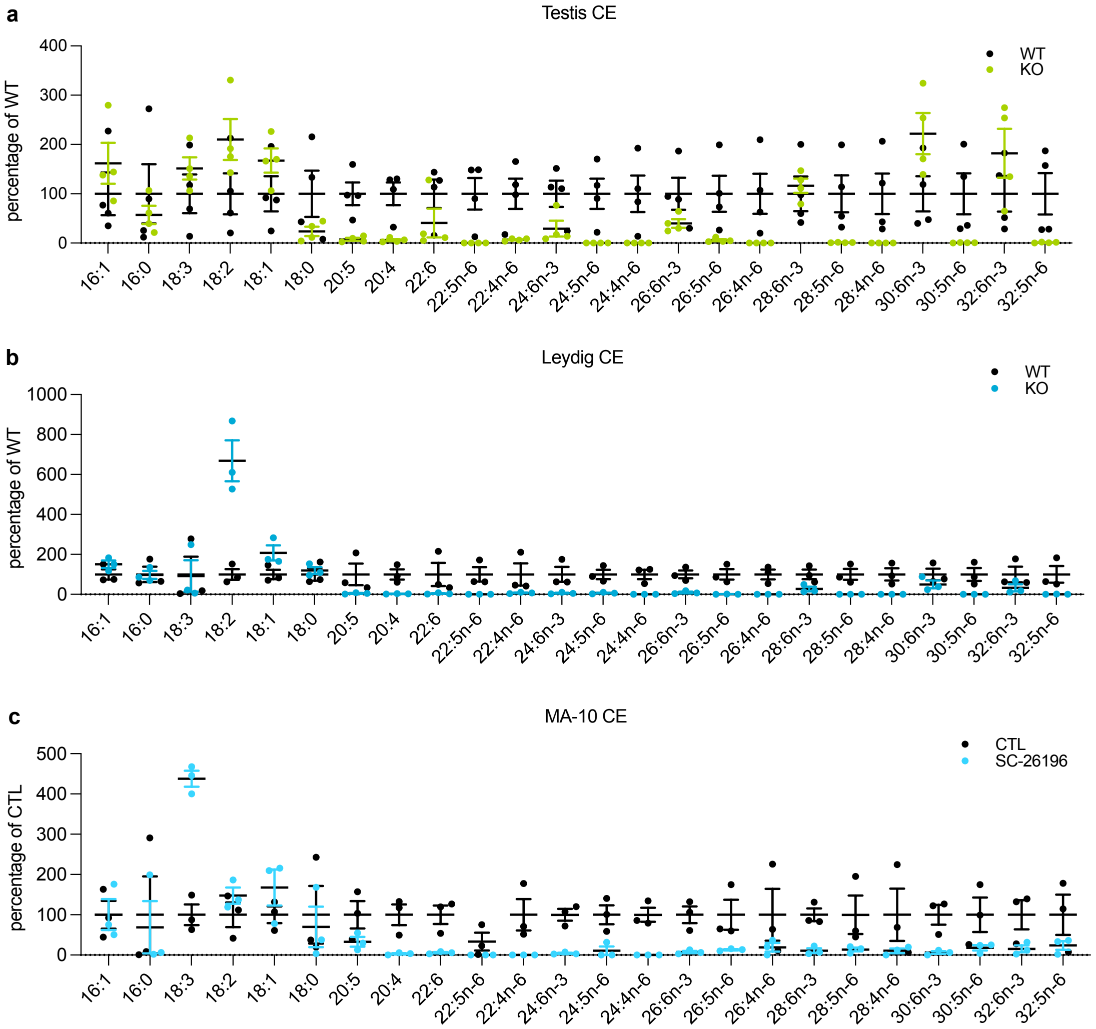

Supplementary Figure 11 Fatty acid molecular species of cholesteryl esters in mouse testes, Leydig cells, and MA-10 cells.

a–**c** Quantification of fatty acid molecular species of cholesteryl esters in testes (**a**), Leydig cells (**b**), and MA-10 cells (**c**). The values of each molecular species were expressed as a percentage relative to WT (wild type; FADS2$^{+/+}$) or CTL (Control). n = 4 (**a**) and n = 3 (**b, c**) for each group. Data shown are the mean ± SEM. KO, knockout (FADS2$^{-/-}$); CE, cholesteryl ester.

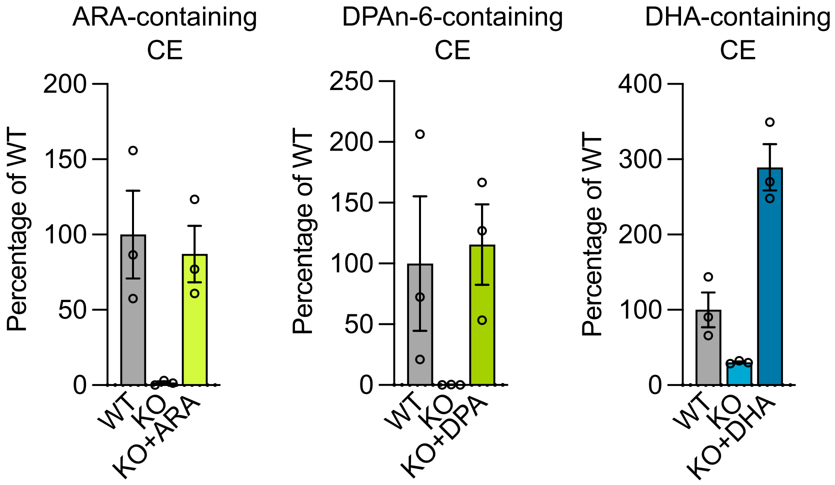

Supplementary Figure 12 Reduced HUFA-containing cholesteryl ester species were replenished by HUFA supplementation.

Quantification of fatty acid molecular species of cholesteryl esters in Leydig cells from FADS2$^{+/+}$ (WT), FADS2$^{-/-}$ (KO), and FADS2$^{-/-}$ mice supplemented with ARA, DPAn-6, or DHA. The values of each molecular species were expressed as a percentage relative to WT. n = 3 for each group. Data shown are the mean ± SEM. CE, cholesteryl ester.

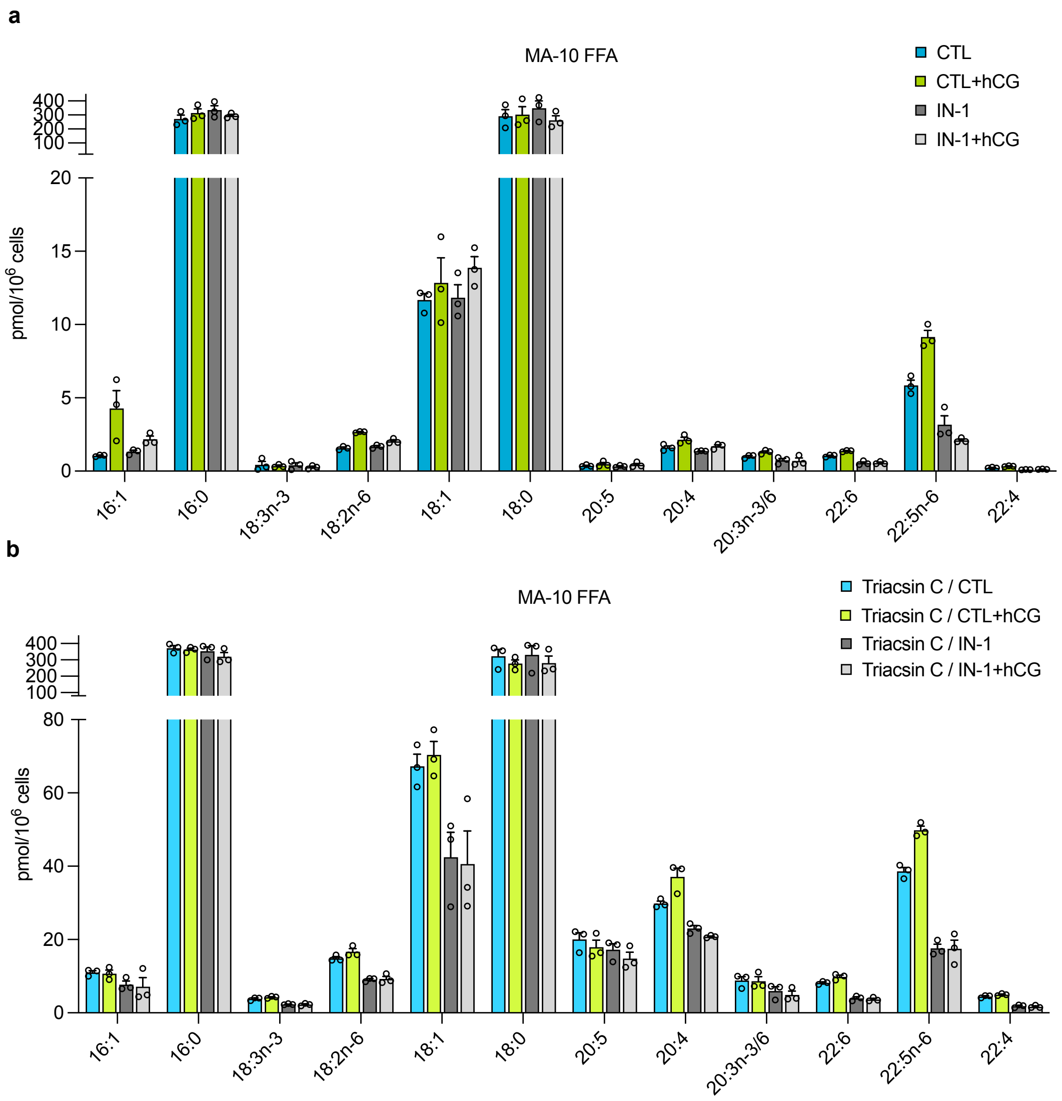

Supplementary Figure 13 HUFA-containing cholesteryl ester species are preferred substrates for HSL.

MA-10 cells were pretreated with DMSO (**a**) or 5 µM Triacsin C (**b**) for 30 min. The cells were then treated with DMSO or 10 µM HSL-IN-1 (HSL inhibitor) for 30 min to evaluate HSL-dependent free fatty acid (FFA) production. Cells were then stimulated with 20 U/mL of hCG for 1 h and harvested for lipid analysis. n = 3 for each group. Data shown are the mean ± SEM. CTL, control; IN-1, HSL-IN-1.

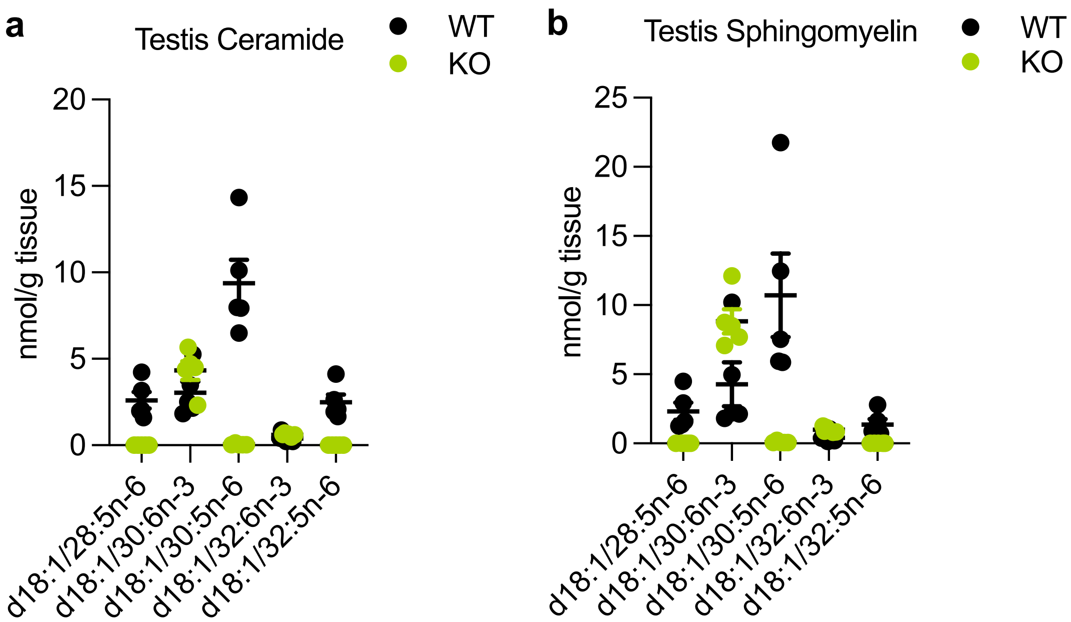

Supplementary Figure 14 Omega-6 very-long-chain-HUFA-containing ceramide and sphingolipid species were depleted from FADS2$^{-/-}$ testis.

Quantification of very-long-chain-HUFA-containing species of ceramide (**a**) and sphingomyelin (**b**) in testes from FADS2$^{+/+}$ (WT) and FADS2$^{-/-}$ (KO) mice. n = 5 for each group. Data shown are the mean ± SEM.

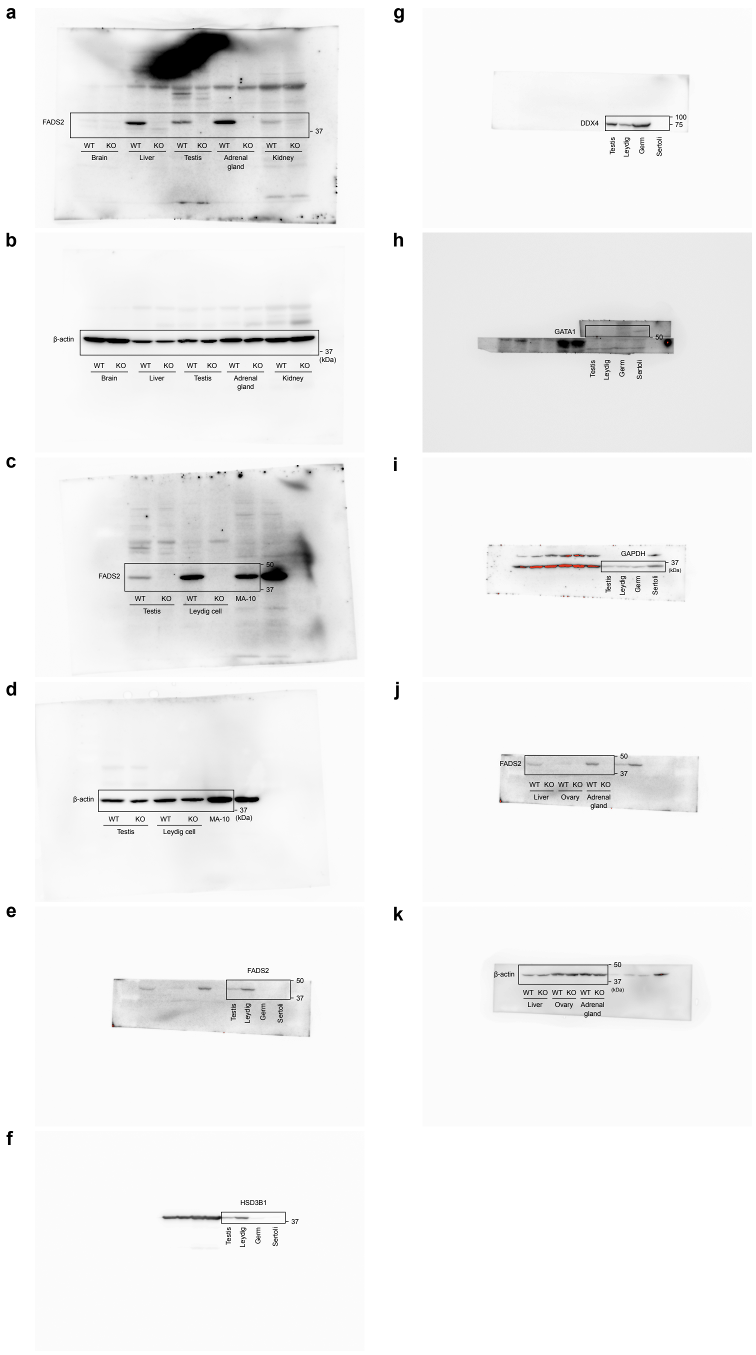

Supplementary Figure 15 Uncropped immunoblot images.

Uncropped immunoblot shown in Fig. 1**b** (**a**, **b**), Fig. 3**a** (**c**, **d**), Supplementary Fig. 2**a** (**e**–**i**), and Supplementary Fig. 2**b** (**j**, **k**).

Supplementary References

1 Saewu, A. *et al.* Primary Sertoli Cell Cultures From Adult Mice Have Different Properties Compared With Those Derived From 20-Day-Old Animals. *Endocrinology* **161**, doi:10.1210/endocr/bqz020 (2020).

2 Zomer, H. D. & Reddi, P. P. Characterization of rodent Sertoli cell primary cultures. *Mol Reprod Dev* **87**, 857-870, doi:10.1002/mrd.23402 (2020).
