# Supplementary material for: Omega-6 highly unsaturated fatty acids in Leydig cells facilitate male sex hormone production
Source: Commun Biol. 2022 Sep 21;5:1001. doi: 10.1038/s42003-022-03972-y (PMC9492697; doi:10.1038/s42003-022-03972-y)
Supplement: Supplementary file 2 — Description of Additional Supplementary Files [file 42003_2022_3972_MOESM2_ESM.pdf]

Description of Additional Supplementary Files

File name: Supplementary Data 1
Description: The source data for graphs in the main figures.

File name: Supplementary Video 1
Description: Motility of sperms from FADS2+/+ mice.

File name: Supplementary Video 2
Description: Motility of sperms from FADS2−/− mice.
