# Supplementary material for: Omega-6 highly unsaturated fatty acids in Leydig cells facilitate male sex hormone production
Source: Commun Biol. 2022 Sep 21;5:1001. doi: 10.1038/s42003-022-03972-y (PMC9492697; doi:10.1038/s42003-022-03972-y)
Supplement: Supplementary file 6 — Reporting Summary [file 42003_2022_3972_MOESM6_ESM.pdf]

nature research

Corresponding author(s): Hyeon-Cheol Lee-Okada

Last updated by author(s): Sep 7, 2022

Reporting Summary

Nature Research wishes to improve the reproducibility of the work that we publish. This form provides structure for consistency and transparency in reporting. For further information on Nature Research policies, see our Editorial Policies and the Editorial Policy Checklist.

Statistics

For all statistical analyses, confirm that the following items are present in the figure legend, table legend, main text, or Methods section.

n/a	Confirmed	
☐	☒	The exact sample size (n) for each experimental group/condition, given as a discrete number and unit of measurement
☐	☒	A statement on whether measurements were taken from distinct samples or whether the same sample was measured repeatedly
☐	☒	The statistical test(s) used AND whether they are one- or two-sided<br>*Only common tests should be described solely by name; describe more complex techniques in the Methods section.*
☐	☒	A description of all covariates tested
☐	☒	A description of any assumptions or corrections, such as tests of normality and adjustment for multiple comparisons
☐	☒	A full description of the statistical parameters including central tendency (e.g. means) or other basic estimates (e.g. regression coefficient) AND variation (e.g. standard deviation) or associated estimates of uncertainty (e.g. confidence intervals)
☐	☒	For null hypothesis testing, the test statistic (e.g. F, t, r) with confidence intervals, effect sizes, degrees of freedom and P value noted<br>*Give P values as exact values whenever suitable.*
☐	☒	For Bayesian analysis, information on the choice of priors and Markov chain Monte Carlo settings
☐	☒	For hierarchical and complex designs, identification of the appropriate level for tests and full reporting of outcomes
☐	☒	Estimates of effect sizes (e.g. Cohen's d, Pearson's r), indicating how they were calculated

Our web collection on statistics for biologists contains articles on many of the points above.

Software and code

Policy information about availability of computer code

Data collection	No software was used.
Data analysis	Statistical analyses were performed using GraphPad Prism 7.

For manuscripts utilizing custom algorithms or software that are central to the research but not yet described in published literature, software must be made available to editors and reviewers. We strongly encourage code deposition in a community repository (e.g. GitHub). See the Nature Research guidelines for submitting code & software for further information.

Data

Policy information about availability of data

All manuscripts must include a data availability statement. This statement should provide the following information, where applicable:

- Accession codes, unique identifiers, or web links for publicly available datasets
- A list of figures that have associated raw data
- A description of any restrictions on data availability

All data generated or analyzed during this study are included in this article and the supplementary information. Raw data for graphs in Fig. 2-5 is available in Supplementary Data 1.

Field-specific reporting

Please select the one below that is the best fit for your research. If you are not sure, read the appropriate sections before making your selection.

☒ Life sciences ☐ Behavioural & social sciences ☐ Ecological, evolutionary & environmental sciences

For a reference copy of the document with all sections, see nature.com/documents/nr-reporting-summary-flat.pdf

Life sciences study design

All studies must disclose on these points even when the disclosure is negative.

Sample size	In accordance with the guidelines of the Animal Research Committee, we attempted to minimize the number of animals used (in most cases, n = 4).
Data exclusions	No data were excluded.
Replication	All attempts to replicate the results were successful.
Randomization	Randomization is not relevant to this study because we determined the experimental groups based on the genotypes of the mice.
Blinding	Blinding is not relevant to this study because the mice we used in this study show apparent phenotypes.

Reporting for specific materials, systems and methods

We require information from authors about some types of materials, experimental systems and methods used in many studies. Here, indicate whether each material, system or method listed is relevant to your study. If you are not sure if a list item applies to your research, read the appropriate section before selecting a response.

Materials & experimental systems

n/a	Involved in the study	
☐	☒	Antibodies
☐	☒	Eukaryotic cell lines
☒	☐	Palaeontology and archaeology
☐	☒	Animals and other organisms
☒	☐	Human research participants
☒	☐	Clinical data
☒	☐	Dual use research of concern

Methods

n/a	Involved in the study	
☒	☐	ChIP-seq
☒	☐	Flow cytometry
☒	☐	MRI-based neuroimaging

Antibodies

Antibodies used	anti-mFADS2 antibody (rabbit, generated in this study), anti-mHSD3B1 antibody (rat, a gift), anti-GATA1 antibody (rat, N6, Santa Cruz Biotechnology), anti-DDX4 antibody (rabbit, 51042-1-AP, Proteintech), anti-GAPDH antibody (mouse, 6C5, Santa Cruz Biotechnology), and anti-β-actin (mouse, AC-15, Santa Cruz Biotechnology)
Validation	The specificity of anti-mFADS2 antibody was validated in this study using FADS2 deficient knockout mouse tissues and cell. Other antibodies used in this study were all validated in the literature and/or on the manufacturer's website.

Eukaryotic cell lines

Policy information about cell lines

Cell line source(s)	MA-10 cells were kindly provided by Dr. Ken-Ichirou Morohashi's lab at Department of Molecular Biology, Graduate School of Medical Sciences, Kyushu University. HEK293T cells were from ATCC.
Authentication	None of the cell lines used were authenticated.
Mycoplasma contamination	The cell lines were not tested for mycoplasma contamination.
Commonly misidentified lines (See ICLAC register)	None.

Animals and other organisms

Policy information about studies involving animals; ARRIVE guidelines recommended for reporting animal research

Laboratory animals	This study only used Fads2+/+, Fads2+/–, and Fads2+/+ mice obtained from heterozygous crossings. Fads2–/– mice were generated from C57BL/6J mice as described previously (FEBS Letters 595 (2021) 1920–1932).
Wild animals	This study did not involve wild animals.
Field-collected samples	This study did not involve samples collected from the field.
Ethics oversight	All animal experiments were approved by and performed in accordance with the guidelines of the Animal Research Committee of the Juntendo University Graduate School of Medicine (approval number: 2020147).

Note that full information on the approval of the study protocol must also be provided in the manuscript.
